# Exploring adhesion forces in biological systems by atomic force microscopy: from nonspecific interactions to molecular recognition

**DOI:** 10.1007/s12551-026-01467-2

**Published:** 2026-09-26

**Authors:** Erick Rafael Dias Rates, Maria do Socorro do Nascimento Amorim, Beatriz da Silva Batista, Álefe Roger Silva França, Rosa Maria Viana Sousa, Leandro Ramos Souza Barbosa, Ralph Santos-Oliveira, Luciana Magalhães Rebêlo Alencar

**Affiliations:** 1 Center for Social Sciences, Health and Technology, Federal University of Maranhão, Advanced Unit, Imperatriz, MA 65915-060 Brazil; 2 Department of Physics, Laboratory of Biophysics and Nanosystems, Federal University of Maranhão, Campus Bacanga, São Luís, MA 65085-580 Brazil; 3 Colégio Universitário (COLUN), Federal University of Maranhão, Campus Bacanga, São Luís, Maranhão 65085-580 Brazil; 4 Department of General Physics, Institute of Physics, University of São Paulo, São Paulo, 05508-000 SP Brazil; 5 Brazilian Nuclear Energy Commission, Nuclear Engineering Institute, Laboratory of Nanoradiopharmacy and Synthesis of New Radiopharmaceuticals, Rio de Janeiro, RJ 21941906 Brazil; 6 University State of Rio de Janeiro, Rio de Janeiro, RJ 20550-013 Brazil

**Keywords:** Atomic force microscopy, Force spectroscopy, Adhesion force, Biological interfaces, Single-cell force spectroscopy, Molecular recognition, Biointerfaces, Viral adhesion

## Abstract

Adhesion is a fundamental biophysical property describing how biological systems establish, maintain, and remodel interactions with their surrounding environment. Force spectroscopy, based on Atomic Force Microscopy (AFM), has emerged as a powerful approach for investigating these nanoscale interactions under physiologically relevant conditions. While existing literature often presents AFM as a general tool for nanomechanics or cellular topography, this review addresses a critical gap by focusing specifically and systematically on biological adhesion as a multidimensional mechanochemical phenomenon. We emphasize that biological adhesion cannot be reduced to a single scalar pull-off force; instead, AFM force-distance curves encode complex biophysical signatures that integrate force magnitude, adhesion energy, interaction range, rupture dynamics, and mechanically mediated detachment processes. In this review, we synthesize the physical foundations of adhesion, including nonspecific physicochemical forces, molecular recognition, polymer dynamics, and mechanical coupling, and demonstrate how these mechanisms translate directly into AFM force curves for cells, tissues, biomaterials, microorganisms, pathogen-modified host cells, and viral particles. We place special emphasis on the contrast between non-functionalized probes, which provide global adhesive fingerprints, and functionalized probes, which enable selected molecular interactions to be probed with piconewton-level sensitivity. Finally, we discuss current limitations and future perspectives regarding experimental standardization, statistical robustness, automated force-curve classification, and multimodal integration. By establishing a unified framework connecting fundamental physical forces, force-curve shape analysis, and biological applications, this review fills an essential gap for researchers seeking to decipher adhesion mechanisms at biological interfaces and within complex living systems.

**Graphical abstract:**

**Figure:**
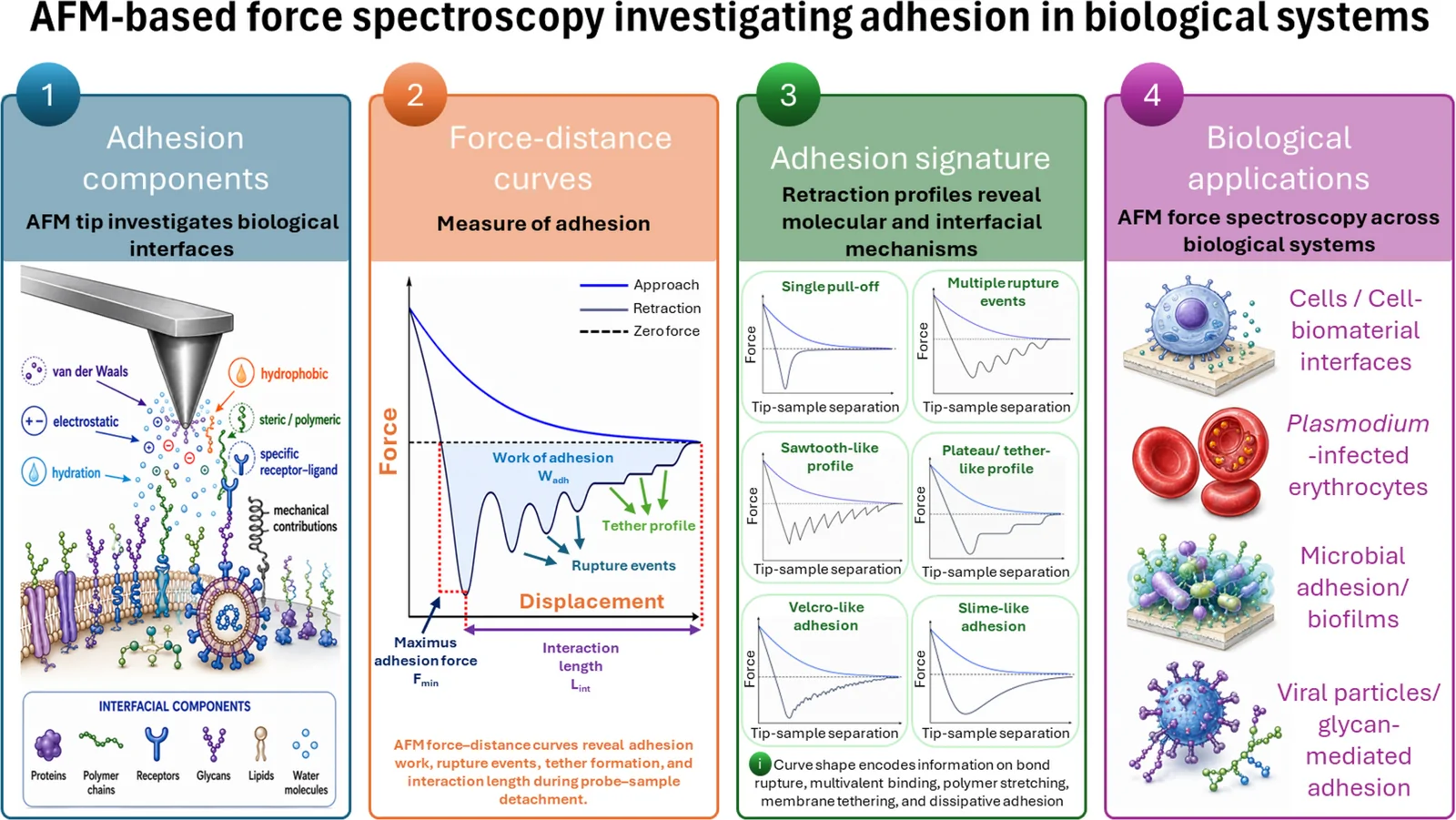

## Introduction

Adhesion is a fundamental biophysical phenomenon that governs how biological systems interact with their surrounding microenvironment. At different length scales, adhesive interactions regulate cell-cell and cell-matrix communication, tissue organization, immune recognition, microbial colonization, virus-host binding, mechanotransduction, and the performance of biomaterials (Delguste et al. 2021; Dufrêne 2015; Harjumäki et al. 2020; Helenius et al. 2008; Viljoen et al. 2021). In biological systems, adhesion is not merely a passive surface property, but a dynamic and highly regulated process that integrates molecular recognition, physicochemical interactions, membrane organization, cytoskeleton coupling, and mechanical feedback (Butt et al. 2005; Friedrichs et al. 2013; Viljoen et al. 2021).

Therefore, understanding adhesion forces is essential to interpreting how cells, microorganisms, viral particles, and biomimetic materials establish, maintain, or disrupt biological interfaces. In addition to its structural and functional role, adhesion can be interpreted as a relevant biophysical parameter for understanding the behavior of biological systems, influencing processes such as cell motility, extracellular matrix remodeling, tumor progression, and cell signaling (Chirasatitsin and Engler 2010; Ingber 2008; Mierke 2024). In this context, the quantification of adhesive forces has been explored as a tool to identify biophysical changes associated with physiological and pathological states. However, the interpretation of this parameter strongly depends on the experimental context and the nature of the interactions involved.

Quantitative investigation of biological adhesion remains a challenge because adhesive interactions are highly heterogeneous and arise from the simultaneous contributions of multiple physicochemical and molecular mechanisms. A single adhesion event can involve nonspecific interactions, defined as universal physical forces present in all potential targets and characterized by low selectivity with respect to the target (such as general electrostatic, hydration, or steric forces), as well as specific interactions, defined as targeted molecular recognition events that occur between distinct bimolecular pairs (such as receptor-ligand pairing, glycan-protein recognition, integrin-extracellular matrix binding, antigen-antibody interactions, and microbial or viral surface adhesins) (Butt et al. 2005; Dufrêne 2015; Harjumäki et al. 2020; Viljoen et al. 2021). Furthermore, biological surfaces are soft, deformable, chemically complex, and frequently covered by dynamic molecular layers, such as the glycocalyx, extracellular polymeric substances, membrane protrusions, or viral glycoprotein shields (Beaussart and El-Kirat-Chatel 2019; Ogharandukun et al. 2020; Viljoen et al. 2021). These characteristics make adhesion forces strongly depend on local surface composition, probe-sample contact geometry (tip radius and effective contact area), loading rate (dF/dt), contact time, environmental conditions (pH, ionic strength, and temperature), and the mechanical state of the biological interface (local stiffness and deformability) (Butt et al. 2005; Friedrichs et al. 2013; Viljoen et al. 2021).

AFM-based force spectroscopy (AFM-FS) offers a unique approach to investigating biological adhesion under physiological conditions, combining nanometer-scale spatial resolution with force sensitivity in the piconewton-to-nanonewton range (Butt et al. 2005; Helenius et al. 2008; Viljoen et al. 2021). By recording force-distance curves, AFM-FS extracts multidimensional mechanical metrics, such as maximum adhesion force, work of adhesion, rupture dynamics, and membrane tether formation, going beyond a simple force value (Butt et al. 2005; Viljoen et al. 2021). Crucially, its experimental versatility allows for various probe configurations, ranging from non-functionalized probes for global physicochemical mapping to functionalized probes, colloidal tips, single cells, and viral particles for analyzing specific molecular interactions (Beaussart and El-Kirat-Chatel 2019; Friedrichs et al. 2013; Harjumäki et al. 2020; Helenius et al. 2008; Ogharandukun et al. 2020), thereby linking nanoscale physical forces to biological function. Despite the widespread use of AFM, the interpretation of adhesion force curves is often handled in a fragmented manner. Many studies report only the maximum adhesion force without fully exploring specific and nonspecific components or parameters such as work of adhesion, interaction extent, rupture distribution, and tether formation patterns. Furthermore, integrated comparisons across cells, tissues, microorganisms, and viral particles from a shared biophysical perspective are rare, limiting the identification of general adhesion patterns and system-specific signatures (Beaussart and El-Kirat-Chatel 2019; Butt et al. 2005; Viljoen et al. 2021).

In this review, we discuss the components of adhesion forces in biological systems from the perspective of AFM-based force spectroscopy. First, we present the physical fundamentals of adhesion and the main forces that contribute to probe-sample interactions in biological environments. We then describe how adhesion is measured from AFM force-distance curves and how experimental parameters influence data interpretation. Subsequently, we review representative applications involving cells, tissues, microorganisms, biomaterials, pathogen-modified cells, and viral particles, with an emphasis on both nonspecific adhesion measured with non-functionalized probes and specific molecular recognition investigated with functionalized probes. Finally, we highlight characteristic adhesion signatures, such as tether formation, multiple rupture events, slime-like behavior, and Velcro-like profiles, discussing how these features can reveal the structure, mechanics, and molecular organization of biological interfaces. By treating adhesion curves as multidimensional signatures rather than isolated force values, this review provides a unified framework for comparing adhesive behavior across cells, tissues, microorganisms, biomaterials, and viral particles.

## Physical basis of adhesion forces in biological systems

**Fig. 1:**
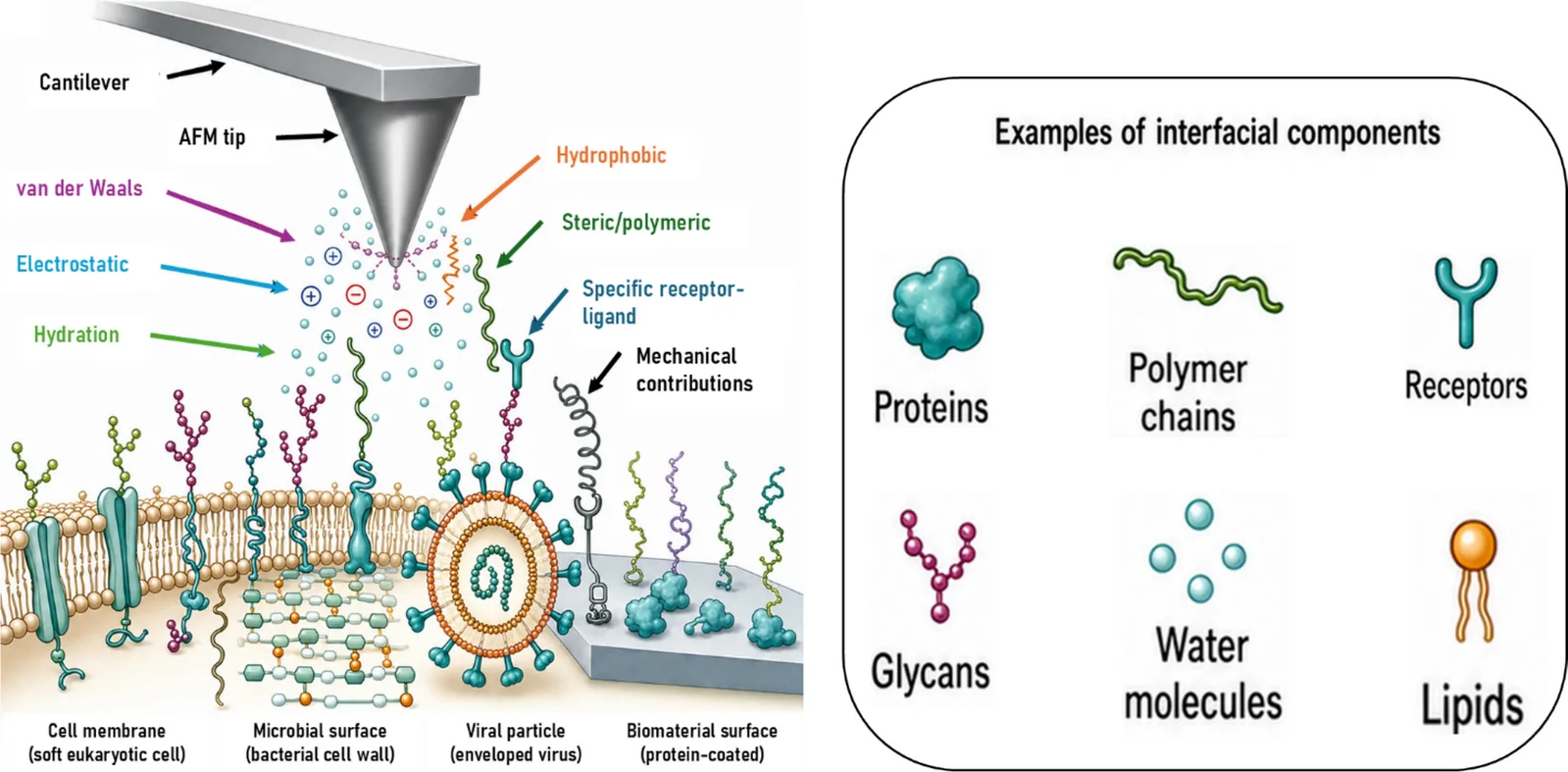
AFM-based adhesion in biological systems. Schematic representation of an AFM tip probing different biological interfaces, including a cell membrane, a microbial surface, a viral particle, and a protein-coated biomaterial. The main contributions to the measured adhesion response are illustrated, including van der Waals, electrostatic, hydration, hydrophobic, steric/polymeric, specific receptor-ligand, and mechanical interactions. Additionally, examples of interfacial components contributing to adhesion are shown, such as proteins, polymer chains, receptors, glycans, water molecules, and lipids

Adhesion in biological systems results from the combined action of physicochemical, molecular, and mechanical interactions operating across different time and length scales. From a physical perspective, adhesion can be understood as the resistance offered by an interface when two bodies previously in contact are separated. Figure 1 summarizes the key components related to adhesion and the parameters derived from the force curves discussed in this section. As schematically illustrated in the figure, the total adhesive response at biological interfaces encompasses a combination of physicochemical, molecular, and mechanical forces.

In biological systems, however, adhesion is rarely the result of a single interaction. Biological interfaces are chemically heterogeneous, mechanically flexible, highly hydrated, and dynamically organized. Cell membranes, bacterial envelopes, extracellular matrices, viral capsids, viral envelopes, and biomaterial surfaces can contain charged groups, hydrophobic domains, glycoproteins, lipids, polysaccharides, proteins, receptors, ligands, and extracellular polymers. Consequently, adhesion strength must be interpreted as an emergent interfacial property arising from the combined, and often coupled, contributions of nonspecific physicochemical forces, specific molecular recognition events, and mechanical contributions associated with the deformation, stretching, unfolding, or detachment of surface structures (Butt et al. 2005; Israelachvili 2011; Viljoen et al. 2021).

A useful conceptual representation is to consider the measured adhesive response as the result of multiple force contributions, as suggested by Eq. 1. This representation is consistent with classical descriptions of intermolecular and surface forces, as well as with approaches used in AFM-FS to interpret adhesive interactions in soft and biological systems (Butt et al. 2005; Israelachvili 2011; Viljoen et al. 2021):

1 $${F}_{measured}={F}_{vdW}+{F}_{elec}+{F}_{hydr}+{F}_{hydroph}+{F}_{steric}+{F}_{spec}+{F}_{mech}+{F}_{diss},$$

where ${F}_{vdW}$ represents van der Waals interactions, ${F}_{elec}$ electrostatic contributions, ${F}_{hydr}$ hydration and solvation forces, ${F}_{hydroph}$ hydrophobic interactions, ${F}_{steric}$ steric or polymer-mediated forces, ${F}_{spec}$ specific molecular recognition events, ${F}_{mech}$ mechanical contributions from the biological interface, and ${F}_{diss}$ dissipative or viscoelastic contributions. Equation 1 should not be interpreted as a universal analytical model or as a simple linear combination of independent forces, but rather as a conceptual framework emphasizing that AFM-measured adhesion emerges from multiple coupled physicochemical, molecular, mechanical, and dissipative contributions.

Nonspecific forces incorporated into Eq. 1 form the physical basis for most biological adhesion measurements. These are interactions common to all potential targets and are generally characterized by low specificity (Müller et al. 2021). Van der Waals forces are universal attractive interactions arising from transient and induced dipoles, becoming relevant when biological surfaces approach each other at nanometer-scale distances. Electrostatic interactions are also central, as biological surfaces commonly contain ionizable groups within phospholipids, proteins, polysaccharides, nucleic acids, and extracellular matrix components. In an aqueous medium, these charges are screened by counter-ions, and the range of electrostatic forces depends on ionic strength, pH, ionic composition, and surface charge density (Butt et al. 2005; Müller et al. 2021).

Hydration, hydrophobic, and steric forces further modulate biological adhesion (Müller et al. 2021). Hydration and solvation forces arise from structured water layers near charged, polar, or chemically organized surfaces and can either prevent intimate contact or stabilize water-mediated interactions. Hydrophobic interactions contribute when nonpolar domains of membranes, proteins, biomaterials, or microbial surfaces are brought into contact in aqueous media, influencing protein adsorption, cell attachment, microbial adhesion, and biomaterial performance. Steric and polymer-mediated forces are especially important in interfaces covered by flexible macromolecular layers, such as the glycocalyx, extracellular polymeric substances, bacterial capsules, mucins, and viral glycoprotein shields. During retraction, these structures may stretch, unfold, detach, or rupture, generating multiple rupture peaks, sawtooth-like profiles, long interaction lengths, or plateau-like regions in force-distance curves (Butt et al. 2005; Israelachvili 2011; Viljoen et al. 2021).

In parallel, specific molecular interactions represent another important class of adhesive contribution in biological systems. These interactions include receptor-ligand binding, antigen-antibody recognition, lectin-glycan interactions, integrin-extracellular matrix binding, biotin-streptavidin interactions, and adhesin-mediated microbial attachment (Müller et al. 2021). Unlike nonspecific forces, specific interactions depend on molecular complementarity, affinity, orientation, accessibility, and local density of binding sites. When AFM probes are functionalized with defined molecules, force spectroscopy can be used to identify and quantify molecular recognition events at the single-molecule or few-molecule level. In this context, the rupture force depends not only on the intrinsic strength of the bond, but also on loading rate, contact time, number of bonds formed, linker flexibility, and mechanical compliance of the biological substrate (Butt et al. 2005; Evans 2001; Evans and Ritchie 1997; Israelachvili 2011; Viljoen et al. 2021). At the single-molecule level, these specific interactions typically operate in the piconewton range, with measured rupture forces depending on the molecular pair, loading rate, linker chemistry, and experimental configuration (Hinterdorfer and Dufrêne 2006).

In microorganisms, adhesion forces are strongly influenced by cell wall architecture, surface proteins, pili, fimbriae, capsules, and extracellular polymeric substances. These structures can generate complex force profiles with multiple rupture events and long interaction distances, reflecting sequential detachment of adhesins, unfolding of surface proteins, extension of pili, or stretching of extracellular polymers (Beaussart and El-Kirat-Chatel 2019; Dufrêne 2015; Mathelié-Guinlet et al. 2019; Sullan et al. 2014). In viral particles, adhesion may involve capsid proteins, envelope glycoproteins, glycans, lipid envelopes, or host-cell receptor mimics (Banerjee and Mukhopadhyay 2016; Casasnovas 2013; Suenaga and Arase 2015). Because viral surfaces can be highly organized and multivalent, AFM force curves may display multiple binding events or long-range adhesive signatures depending on the molecular architecture of the viral interface and the way it is mechanically sampled during retraction (Delguste et al. 2021; Ogharandukun et al. 2020). In glycan-rich viral or virus-like systems, force curves may display characteristic slime-like or Velcro-like profiles, reflecting rate-dependent sampling of glycan accessibility, multivalent contacts, and dissipative detachment processes (Ogharandukun et al. 2020).

Consequently, the magnitude of biological adhesion spans several orders of magnitude depending on the system’s scale and complexity. While single viral glycoprotein-receptor or individual receptor-ligand interactions generate forces in the piconewton regime (Cardoso-Lima et al. 2023; Hinterdorfer and Dufrêne 2006), whole microbial adhesion, cell attachment, and extracellular matrix interactions involve multivalent cooperative binding and structural compliance that can extend into the piconewton-to-nanonewton range (Aguayo et al. 2015; Yakovenko et al. 2008).

Taken together, adhesion forces in biological systems should be interpreted as emergent and context-dependent interfacial properties, rather than isolated numerical values. The measured adhesive response depends on the chemical nature of the interacting surfaces, the presence of hydrated or polymeric surface layers, and the dynamics of bond formation and rupture (Butt et al. 2005; Evans 2001). Crucially, modern AFM-FS is uniquely suited to probe biological adhesion across multiple hierarchical levels, from sub-nanonewton single-molecule unbinding events (Dufrêne 2015) to whole-cell detachment forces that commonly reach the nanonewton regime (Beaussart and El-Kirat-Chatel 2019), by selecting cantilevers with spring constants appropriate for each force range, typically ~ 0.01–0.1 N/m for single-molecule force spectroscopy and up to several N/m for high-force cellular or tissue measurements (Müller et al. 2009). This complexity is precisely what gives AFM-FS the ability to analyze not only the magnitude of adhesion, but also the shape, extent, energy, and pattern of rupture through force-distance curves, making it possible to relate nanoscale interactions to biological structure and function. The following section expands on this physical basis by discussing how AFM force-distance curves are obtained, processed, and interpreted to extract adhesion-related parameters and biophysical signatures of biological systems.

## AFM force-distance curves: extracting adhesion parameters and biophysical signatures

After establishing the physical basis of adhesion, the next step is to understand how AFM force-distance curves are acquired, processed, and interpreted to extract biologically significant information. In AFM-FS, cantilever deflection recorded during approach and retraction is converted into force using the calibrated spring constant, while the piezoelectric displacement is converted into tip-sample separation after correcting for cantilever bending. The resulting force-distance curve provides a local mechanical and adhesive fingerprint of the probed interface, integrating contributions from surface chemistry, molecular recognition, sample deformation, and dissipative processes (Butt et al. 2005; Friedrichs et al. 2013).

Although the force ${F}_{adh}$, remains the most frequently reported parameter in AFM-based adhesion studies, it should not be interpreted in isolation. As discussed above, biological interfaces may exhibit similar detachment forces but different retraction profiles, interaction lengths, adhesive energies, and rupture patterns. For this reason, a more informative analysis combines the magnitude of the force with indicators that capture how detachment occurs over distance and time. These indicators include adhesion work, the persistence of adhesive interactions during retraction, the number and distribution of rupture events, and the presence of mechanically mediated signatures such as tethers, plateaus, or dissipative tails (Butt et al. 2005; Viljoen et al. 2021).

**Fig. 2:**
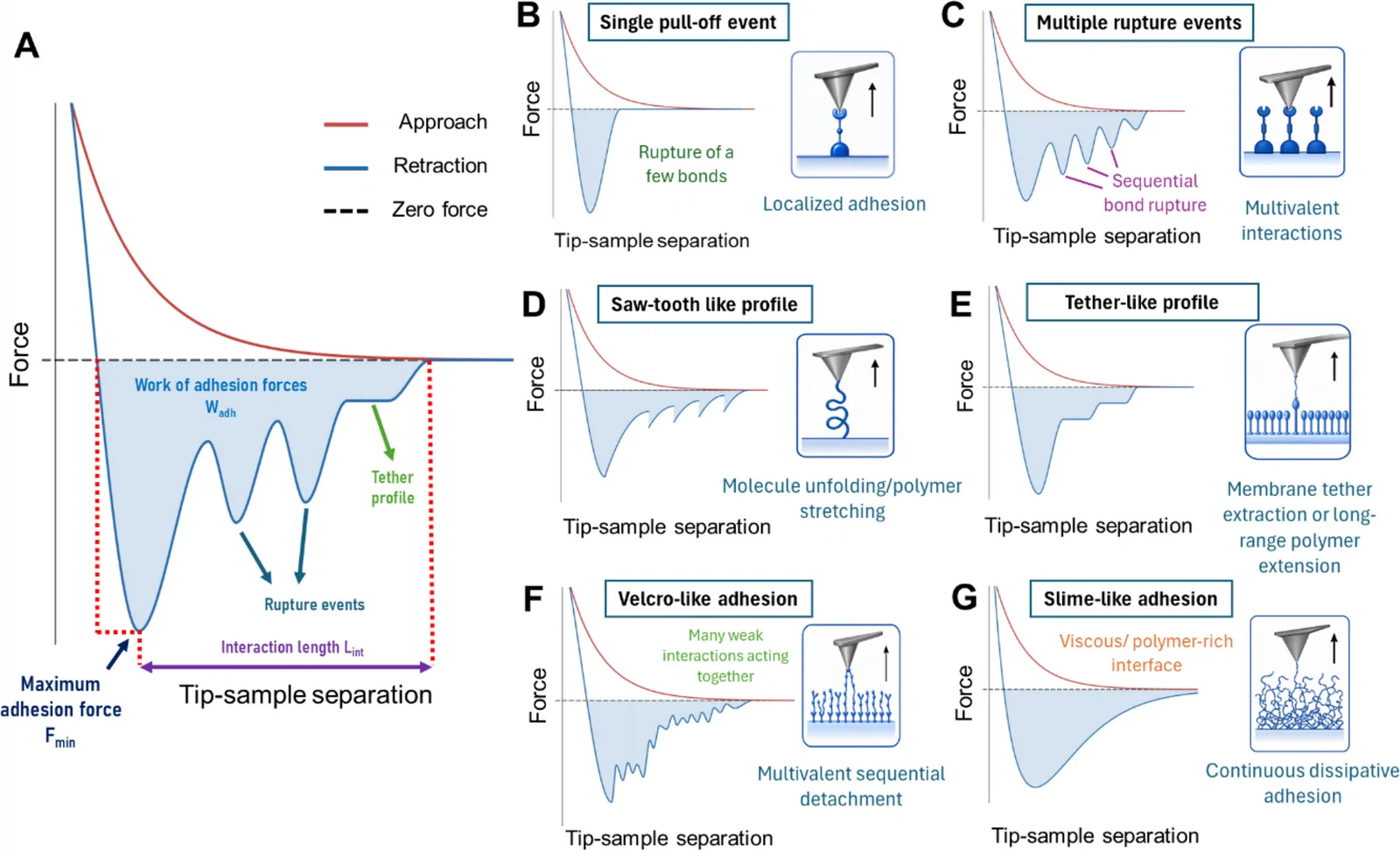
Representative adhesion signatures in AFM force spectroscopy of biological systems. **A** Simplified AFM force-distance curve highlighting the main adhesion-related parameters obtained during force spectroscopy. The approach curve is shown in red and the retraction curve in blue. The most negative force during retraction corresponds to the adhesion force (F_adh_), the shaded area under the retraction curve below zero force represents the work of adhesion (W_adh_), and the distance over which adhesive interactions persist defines the interaction length (L_int_). The retraction segment of AFM force-distance curves can display distinct adhesive profiles that reflect different molecular and mechanical processes at biological interfaces. **B** A single pull-off event is characterized by one dominant rupture peak followed by rapid return to zero force, suggesting localized adhesion or rupture of a small number of bonds. **C** Multiple rupture events indicate sequential detachment processes that may arise from heterogeneous binding sites, multivalent interactions, or successive molecular bond rupture. **D** Sawtooth-like profiles are commonly associated with macromolecule unfolding, polymer stretching, or sequential rupture of modular structures. **E** Plateau or tether-like profiles show an extended negative-force region, consistent with membrane tether extraction, long-range polymer extension, or detachment of surface-associated structures. **F** Velcro-like adhesion is characterized by many closely spaced rupture events over an extended interaction length, reflecting multivalent detachment mediated by numerous weak interactions. **G** Slime-like adhesion appears as a broad and smooth adhesive region with gradual detachment, suggesting a viscous, polymer-rich, hydrated, or highly dissipative interface. These schematic profiles represent idealized adhesion signatures; in real biological systems, mixed or overlapping features may occur depending on probe chemistry, loading conditions, contact geometry, environmental conditions, and sample heterogeneity. Together, these signatures illustrate that AFM retraction curves provide biophysical information beyond the maximum adhesion force, revealing molecular rupture, multivalent binding, polymer stretching, membrane tethering, and dissipative adhesion

The force-distance curve and its representative retraction profiles, along with their possible biological interpretations, are summarized in Fig. 2. The curve provides distinct information from the AFM-FS experiment (Fig. 2A), including the approach and retraction segments (red and blue lines, respectively), the contact region, the maximum adhesion force (${F}_{adh}$), the work of adhesion (${W}_{adh}$), the interaction length (${L}_{int}$), and representative rupture events.

Operationally, the adhesion force ${F}_{adh}$ is extracted from the minimum force value observed during the retraction segment of the force curve (Fig. 2A), as shown in Eq. 2 (Butt et al. 2005):

2 $${F}_{adh}=\left|{F}_{min}\right|,$$

where $\left|{F}_{min}\right|$ represents the most negative force recorded during probe withdrawal. Although simple and experimentally accessible ${F}_{adh}$ represents only one aspect of the adhesive interaction. A complementary parameter is the work of adhesion ${W}_{adh}$, estimated from the integral of the adhesion region in the retraction curve (Fig. 2A) using Eq. 3 (Butt et al. 2005):

3 $${W}_{adh}= {\int}_{{z}_{1}}^{{z}_{2}}\left|{F}_{ret}(z)\right|dz \mathrm{f}\mathrm{o}\mathrm{r} {F}_{ret}\left(z\right)<0,$$

where ${z}_{1}$ and ${z}_{2}$ define the adhesive region of the curve and ${F}_{ret}\left(z\right)$ represents the negative-force portion of the retraction curve. Whereas ${F}_{adh}$ describes the maximum detachment force, ${W}_{adh}$ provides information on the total energy required to separate the probe from the biological interface. This distinction is particularly important in soft and polymer-rich biological systems, where long-range interactions, membrane tethers, or extracellular macromolecules can produce extended adhesive profiles with high energy dissipation even when the maximum pull-off force is moderate (Butt et al. 2005; Viljoen et al. 2021).

A discrete single-step detachment event (Fig. 2B) represents a single-step adhesion signature in force spectroscopy, typically associated with the rupture of an individual molecular bond or a small set of highly synchronized cooperative interactions under low peak compressive loads (i.e., low indentation forces that limit sample deformation and contact area expansion) (Baumgartner et al. 2000; Hinterdorfer et al. 1996). When the probe detaches from more heterogeneous or multivalent interfaces, sequential separation can generate multiple discrete disruption events (Fig. 2C). These independent peaks reflect the consecutive disruption of different molecular bridges as tension is redistributed in the contact zone, a characteristic behavior of adhesion systems in cell membranes and receptor-ligand interactions involving multiple sites on biological surfaces (Alsteens et al. 2015; Dufrêne 2015).

To connect these measured rupture forces to underlying energetic and kinetic properties, dynamic force spectroscopy (DFS) investigates the dependence of rupture force on the loading rate. Molecular bonds are dynamic; thus, measured rupture forces increase as mechanical stress is applied more rapidly (r=dF/dt) because the bond has less time to undergo thermal dissociation. The Bell-Evans model is widely used to describe this behavior, as expressed in Eq. 4 (Björnham and Andersson 2017; Evans 2001; Evans and Ritchie 1997):

4 $${F}^{*}=\frac{{k}_{B}T}{{x}_{\beta }}\mathrm{ln}\left(\frac{{r}_{f}{x}_{\beta }}{{k}_{B}T{k}_{off}^{0}}\right),$$

where ${F}^{*}$ is the most probable rupture force, ${k}_{B}$ is the Boltzmann constant, T is the absolute temperature, ${x}_{\beta }$ is the distance from the bound state to the transition state, ${k}_{off}^{0}$ is the spontaneous dissociation rate in the absence of force, and ${r}_{f}$ is the loading rate. DFS has been widely applied to characterize receptor-ligand kinetics, protein unfolding, microbial adhesins, and viral entry complexes (Björnham and Andersson 2017; Evans 2001; Evans and Ritchie 1997).

While dynamic force spectroscopy evaluates discrete, single-molecule kinetic properties under applied mechanical force, describing adhesion across finite contact interfaces requires continuous contact mechanical models. In this context, classical continuum models such as Johnson–Kendall–Roberts (JKR) and Derjaguin–Muller–Toporov (DMT) help relate measured pull-off forces to probe geometry, interfacial surface energy, and sample mechanics (Derjaguin et al. 1975; Johnson et al. 1971). However, direct application of JKR or DMT to biological systems demands caution. Cells, tissues, microorganisms, and viral particles are soft, viscoelastic, rough, hydrated, and molecularly heterogeneous, often deviating from ideal elastic continuum assumptions. Therefore, contact mechanical models should be used critically and only when experimental conditions match their underlying assumptions (Butt et al. 2005; Viljoen et al. 2021).

A remarkably distinct pattern is the sawtooth-shaped profile (Fig. 2D), characterized by a sequence of gradual increases in force followed by abrupt drops in force. This mechanical signature arises from the progressive, domain-by-domain unfolding of modular macromolecules, where each unfolding event corresponds to the rupture of an individual protein domain, leading to a sudden increase in contour length. This behavior constitutes a well-established mechanical signature of SMFS in polyproteins and modular protein architectures (Hoffmann and Dougan 2012; Hughes and Dougan 2016). On the other hand, when highly deformable cell membranes or lipid systems are investigated, the force-extension curve often transitions to a plateau or tether-like profile (Fig. 2E). This constant force regime reflects the formation and elongation of membrane tethers, in which lipid material is continuously extracted from a membrane reservoir, resulting in a balance of forces governed by membrane tension and bending energy until eventual rupture or detachment of the cable (Helenius et al. 2008; Krieg et al. 2018; Sun et al. 2005).

In densely functionalized or highly multivalent adhesive interfaces, force signatures can become increasingly cooperative, reflecting the simultaneous contribution of multiple specific and nonspecific interactions acting in parallel. Velcro-like profiles (Fig. 2F) are phenomenological descriptors characterized by a dense, partially overlapping sequence of rupture events distributed over large distances (Khan et al. 2022; Ogharandukun et al. 2020). This behavior has been described in glycan-rich viral systems, where mechanical sampling of the glycan shield can produce velocity-dependent, Velcro-like adhesion profiles (Ogharandukun et al. 2020). Conceptually, similar multivalent detachment responses occur at various biological interfaces, including microbial and cellular adhesion, where pili, fimbriae, adhesins, capsules, or extracellular polymeric substances can generate multiple contact points and sequential rupture events under mechanical load (Dufrêne 2015; Khan et al. 2022; Wang et al. 2025; Xiao and Dufrêne 2016). Importantly, these signatures are not mutually exclusive; depending on kinetic parameters such as indentation speed and contact time, a single biological interface can transition between continuous viscous detachment and discrete Velcro-like rupture profiles (Ogharandukun et al. 2020).

Slime-like adhesion, as schematically represented in Fig. 2G, is characterized by a broad, smooth, and highly dissipative negative-force region with gradual detachment rather than discrete rupture events (Ogharandukun et al. 2020). Depending on the biological system, probe configuration, and environmental conditions, similar profiles may arise from viscoelastic shedding, polymer-rich interfaces, hydrated extracellular matrices, extracellular polymeric substances, glycocalyx layers, mucosal components, or viscous bridge-like effects (Fang et al. 2000; Ogharandukun et al. 2020). In these cases, the adhesive response is dominated less by the rupture of isolated molecular bonds and more by collective dissipation, macromolecular relaxation, and gradual separation of soft hydrated interfacial layers (Abu-Lail and Camesano 2003; Fang et al. 2000; Sotres and Arnebrant 2013).

The work of adhesion is particularly useful for distinguishing localized detachment from distributed or long-range adhesive interactions. A sharp rupture peak may produce a high maximum adhesion force but a relatively small adhesive energy, whereas a broad tether-like, polymer-mediated, or dissipative profile may display a moderate force peak but a large work of adhesion. This distinction is especially relevant for soft and polymer-rich biological interfaces, such as cells with developed glycocalyx layers, bacterial surfaces covered by extracellular polymeric substances, viral envelopes containing flexible glycoproteins, and hydrated biomaterials with adsorbed proteins or polymer chains. In these cases, the area under the adhesive portion of the retraction curve can better reflect the energetic cost of separation than the maximum pull-off force alone (Butt et al. 2005; Viljoen et al. 2021).

Interaction length provides complementary information because it reflects the distance over which adhesive interactions persist during probe withdrawal. Short interaction lengths are usually associated with localized rupture events, whereas long interaction lengths may indicate stretching of surface polymers, unfolding of macromolecules, extraction of membrane tethers, or sequential rupture of multiple adhesive bonds. In biological samples, this parameter is particularly sensitive to membrane organization, polymer density, extracellular matrix composition, microbial appendages, and viral glycoprotein architecture. Thus, interaction length should be interpreted as a structural and mechanical descriptor of the interface, rather than as a purely geometric feature of the force curve (Dufrêne 2015; Sun et al. 2005).

**Fig. 3:**
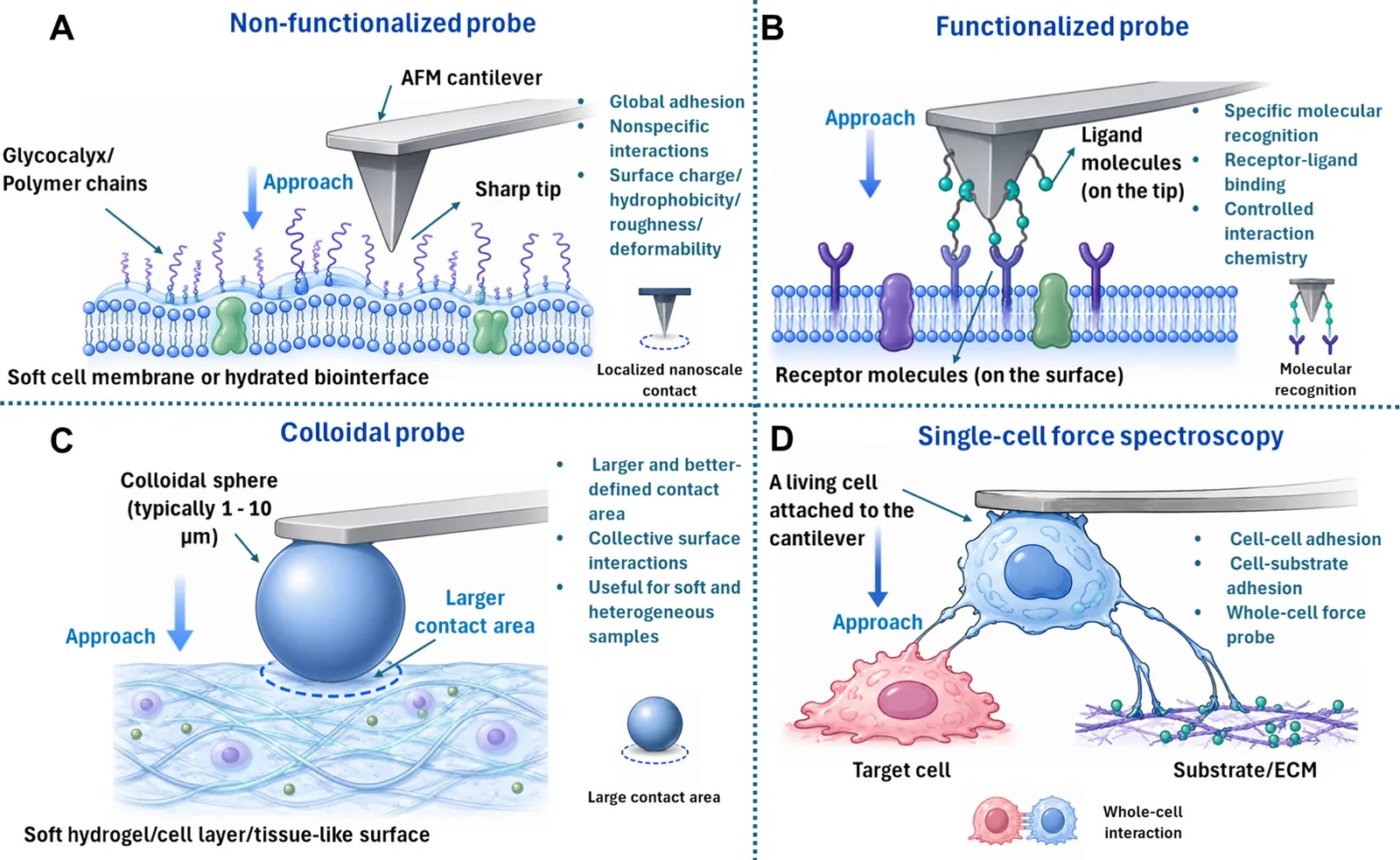
AFM probe configurations for adhesion measurements in biological systems. The configuration of the AFM probe strongly influences the contact geometry, interaction scale, and biological interpretation of adhesion measurements. **A** Non-functionalized sharp probes provide global adhesive fingerprints of biological interfaces, integrating contributions from nonspecific interactions, surface charge, hydration, hydrophobicity, roughness, deformability, and polymeric surface layers. **B** Functionalized probes contain defined ligands, receptors, antibodies, peptides, glycans, or other molecules attached to the tip through linker chemistries, enabling the detection of specific molecular recognition events such as receptor-ligand binding. **C** Colloidal probes replace the sharp tip with a spherical particle, providing a larger and better-defined contact area that is useful for probing collective adhesive responses in soft tissues, hydrogels, cell layers, biomaterials, and other heterogeneous surfaces. **D** In single-cell force spectroscopy, an entire living cell is attached to the cantilever and used as a force probe to measure integrated cell-cell or cell-substrate adhesion under controlled contact conditions. Together, these configurations illustrate that AFM-based adhesion measurements must be interpreted according to the probe geometry, functionalization strategy, contact area, and biological scale being investigated

The interpretation of adhesion curves also depends strongly on the probe configuration (Fig. 3). Non-functionalized probes (Fig. 3A) are useful for obtaining global adhesive fingerprints of biological surfaces because they capture the combined influence of surface charge, hydration, hydrophobicity, roughness, deformability, and nonspecific probe-sample interactions. These measurements are valuable for comparing different biological or material conditions, such as treated and untreated cells, microbial strains, viral particles, or biomaterial surfaces. However, because several interaction mechanisms contribute simultaneously, adhesion measured with non-functionalized probes should generally be interpreted as an integrated interfacial response rather than as evidence of a specific molecular pair (Beaussart and El-Kirat-Chatel 2019; Dufrêne 2015; Viljoen et al. 2021).

Functionalized probes (Fig. 3B) are used when the goal is to investigate specific molecular recognition events. By attaching ligands, antibodies, peptides, lectins, extracellular matrix proteins, glycans, or receptor fragments to the AFM tip, force spectroscopy can probe selected interactions at the single-molecule or few-molecule level. In this configuration, however, interpretation depends critically on linker chemistry, ligand density and orientation, tip passivation, contact time, loading rate, and suppression of nonspecific adhesion. Specificity should therefore be supported by appropriate controls, including blocking assays, competition experiments, enzymatic treatments, receptor inhibition, and comparison with non-functionalized or control-functionalized probes. When these controls are properly implemented, functionalized AFM probes can transform adhesion measurements from global interfacial fingerprints into molecular recognition assays (Evans 2001; Harjumäki et al. 2020; Müller et al. 2021).

Colloidal probes (Fig. 3C) and SCFS (Fig. 3D) expand the experimental possibilities of AFM-based adhesion measurements by changing the geometry and biological meaning of the contact. Colloidal probes provide a better-defined and larger contact area than sharp tips, making them useful when adhesion depends on collective interactions over soft tissues, cells, hydrogels, polymer films, or biomaterial surfaces. In single-cell force spectroscopy, an entire living cell is immobilized on the cantilever and used as the force probe, allowing direct measurement of cell-cell or cell-substrate adhesion under controlled contact force, contact time, and retraction conditions. These approaches are particularly valuable when the biological question involves integrated surface responses rather than highly localized nanoscale contacts (Ducker et al. 1991).

Beyond conventional functionalization strategies, Fluidic Force Microscopy (FluidFM) has emerged as a powerful adaptation of AFM-FS for SCFS. By integrating hollow cantilevers connected to microfluidic channels, FluidFM replaces chemical glues with reversible, pressure-driven cell trapping (Guillaume-Gentil et al. 2014). Applying pressure gently fixes individual microorganisms or mammalian cells to the probe aperture, enabling whole-cell detachment measurements over a remarkably broad force regime (~ 500 pN to 1.5 µN) and allowing serial adhesion measurements of hundreds of individual cells with a single probe (Guillaume-Gentil et al. 2014). For instance, Challa et al. (2023) integrated FluidFM-based SCFS with traction force microscopy to correlate vertical adhesive forces with basal contractile forces in HeLa cells, demonstrating that actomyosin contractility can be partially independent of vertical detachment resistance.

Force-volume mapping and quantitative imaging modes add spatial resolution to AFM-based adhesion analysis. Instead of interpreting a single force-distance curve as representative of the entire sample, arrays of curves can be acquired over defined regions to generate maps of adhesion force, stiffness, deformation, dissipation, and other local mechanical parameters. This approach is particularly useful for biological interfaces because adhesion is rarely homogeneous. In cells, force maps can reveal spatial heterogeneities in mechanical and adhesive properties across different cellular regions, reflecting variations in local structure, composition, and cytoskeletal organization (Dufrêne et al. 2013; Kerdegari et al. 2023; Krieg et al. 2018). In microorganisms and biofilms, they can identify adhesive heterogeneity related to cell wall architecture, surface proteins, capsules, or extracellular polymeric substances (Dufrêne 2014; Xiao and Dufrêne 2016). In viral particles or virus-like structures, spatially resolved measurements can help correlate topography, local stiffness, and adhesive signatures (Cardoso-Lima et al. 2022; De Araujo Dorneles et al. 2022). Thus, adhesion mapping provides a bridge between structural imaging and quantitative force spectroscopy, allowing nanoscale mechanical and adhesive properties to be interpreted in their spatial biological context.

The reliability of AFM-based adhesion measurements depends on rigorous experimental control. Instrumental factors such as cantilever spring constant, deflection sensitivity, tip radius, probe chemistry, loading force, contact time, retraction velocity, and sampling rate can directly influence the shape and magnitude of force-distance curves. Environmental conditions, including buffer composition, ionic strength, pH, temperature, and hydration state, also affect both nonspecific and specific interactions. In biological systems, additional sources of variability arise from cell viability, passage number, membrane integrity, extracellular matrix adsorption, microbial growth phase, biofilm maturity, sample immobilization, and viral particle preservation. Because adhesion is highly context-dependent, detailed reporting of these conditions is essential for reproducibility and for meaningful comparison among studies (Amorim et al. 2022; Dufrêne et al. 2017; Friedrichs et al. 2013).

Data processing is equally important for the reliable interpretation of adhesion signatures (Butt et al. 2005; Friedrichs et al. 2013; Viljoen et al. 2021). After acquisition, force-distance curves require baseline correction, contact point determination, conversion of cantilever deflection into force, and correction of piezoelectric displacement into tip-sample separation. In adhesion studies, additional care is needed to identify rupture events, distinguish true adhesive interactions from artifacts, and define objective criteria for curve inclusion or exclusion. Biological force curves are often heterogeneous and may contain failed contacts, nonspecific sticking, drift, hydrodynamic contributions, or mechanical instabilities. Therefore, analysis should be based on a sufficiently large number of curves, transparent preprocessing steps, appropriate statistical treatment, and, whenever possible, reporting of full parameter distributions. Presenting distributions of adhesion force, work of adhesion, interaction length, and rupture events is generally more informative than reporting only mean values, especially when biological heterogeneity is part of the phenomenon under investigation.

AFM force-distance curves should be interpreted as multidimensional fingerprints of biological interfaces (Butt et al. 2005; Dufrêne et al. 2017; Sun et al. 2005). The maximum adhesion force identifies the greatest resistance to detachment, whereas the work of adhesion reflects the energetic cost of separation, and the interaction length indicates how far adhesive interactions persist during probe withdrawal. In turn, the rupture pattern provides insight into the molecular and mechanical processes involved in detachment, including localized bond rupture, multivalent binding, polymer stretching, membrane tether extraction, or dissipative adhesion. By combining these descriptors with probe functionalization, spatial force mapping, rigorous controls, and appropriate statistical analysis, AFM force spectroscopy can move beyond simple adhesion quantification and reveal how biological surfaces organize, interact, deform, and respond at the nanoscale.

The following sections build on this methodological framework by examining how AFM-based adhesion measurements have been applied to representative biological systems, including cells, pathogen-modified cells, microorganisms, biomaterials, and viral particles. Emphasis is placed on how nonspecific adhesion, specific molecular recognition, probe configuration, and force-curve signatures have been used to connect nanoscale adhesion measurements with biological mechanisms.

## State of the art: AFM-based adhesion measurements in biological systems

**Fig. 4:**
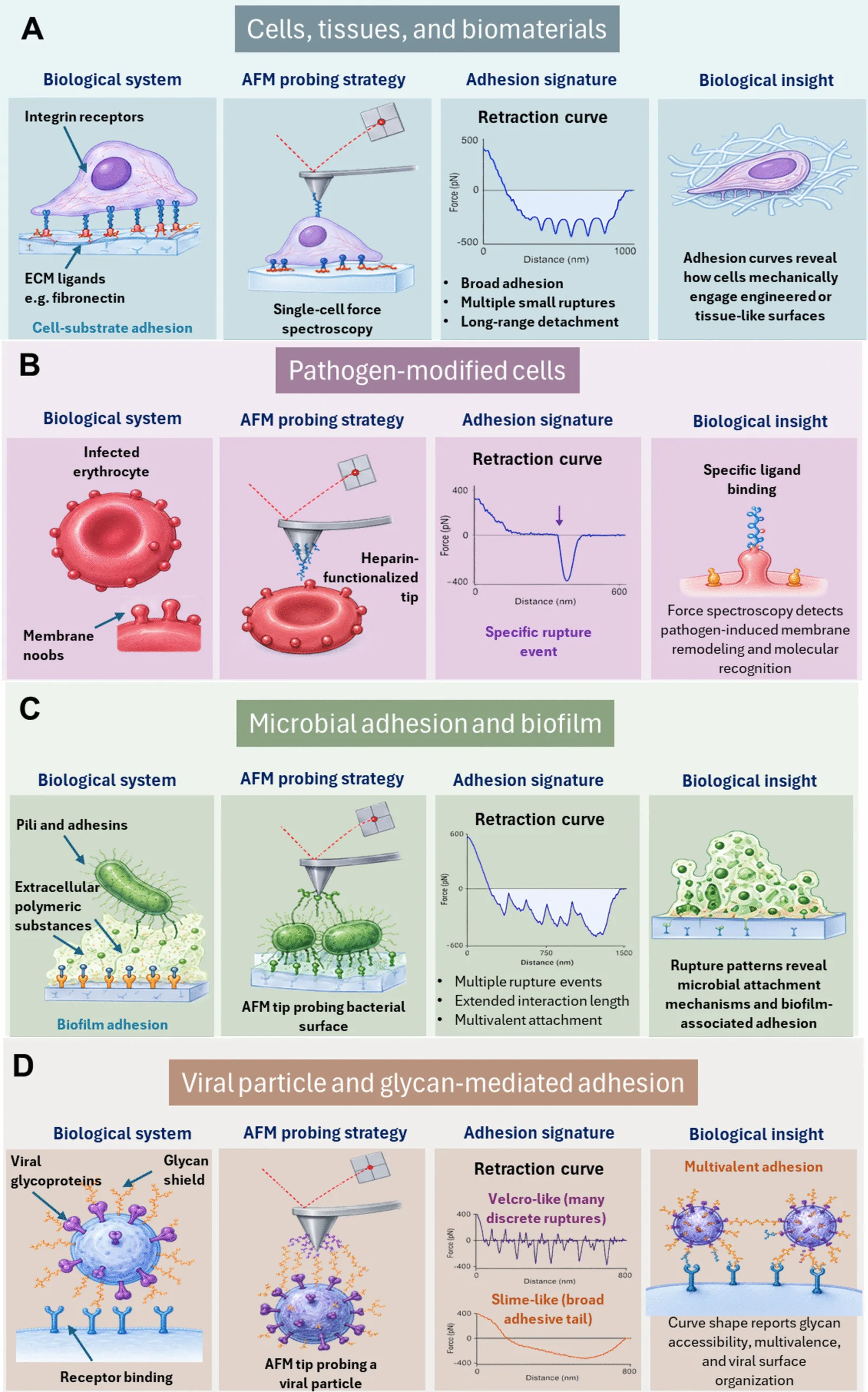
AFM-based adhesion studies across biological systems. Schematic overview illustrating how AFM-based force spectroscopy connects biological interfaces, probing strategies, adhesion-curve signatures, and mechanistic interpretation. **A** In cells, tissues, and biomaterial interfaces, AFM-based approaches, including single-cell force spectroscopy and functionalized substrates, can reveal cell-substrate adhesion, integrin-mediated recognition, and biomaterial-dependent adhesive responses (Friedrichs et al. 2013; Harjumäki et al. 2020; Helenius et al. 2008; Zeng et al. 2023). **B** In pathogen-modified cells, such as Plasmodium falciparum-infected erythrocytes, functionalized probes can detect parasite-induced membrane remodeling, knob formation, and specific ligand binding events (Aikawa et al. 1996; Nagao et al. 2000; Valle-Delgado et al. 2013). **C** In microbial systems and biofilms, adhesion curves can reveal the contribution of pili, adhesins, extracellular polymeric substances, surface roughness, and multivalent attachment to complex rupture patterns (Da Costa et al. 2022; Dufrêne 2015; Feuillie et al. 2018; Formosa-Dague et al. 2016; Sullan et al. 2014). **D** In viral particles and glycan-rich interfaces, force spectroscopy can resolve receptor binding, glycoprotein accessibility, glycan-mediated multivalence, and characteristic slime-like or Velcro-like adhesion profiles (Delguste et al. 2021; Ogharandukun et al. 2020; Simpson et al. 2023; Yang et al. 2020). Together, these examples emphasize that AFM retraction curves provide information beyond maximum adhesion force, acting as multidimensional biophysical signatures of molecular recognition, surface organization, mechanical coupling, and dissipative behavior

This section reviews representative peer-reviewed studies in which AFM-based force spectroscopy was applied to investigate adhesion in biological systems. The selected examples include living cells, cancer cells, tissues, cell-biomaterial interfaces, pathogen-modified cells, microbial adhesion, biofilms, viral particles, and glycan-mediated adhesion. Rather than providing an exhaustive survey, this section emphasizes studies in which adhesion parameters, force-curve signatures, or spatial adhesion maps were directly connected to biological mechanisms, such as molecular recognition, surface remodeling, multivalent binding, tether formation, dissipative detachment, or biomaterial response. Figure 4 provides a schematic overview of these representative applications, illustrating how AFM probing strategies and adhesion signatures can be linked to biological interpretation across different systems.

### Cells, cancer cells, cell-biomaterial interfaces, and tissues

SCFS has become a central AFM-based strategy for quantifying cell adhesion because it allows living cells to be used as force probes under controlled contact conditions. Helenius et al. (2008) described how cells attached to AFM cantilevers can be brought into contact with other cells or substrates to measure cell-cell and cell-substrate adhesion while controlling contact force, contact time, and retraction conditions. Friedrichs et al. (2013) subsequently provided a practical methodological framework for quantifying the adhesive strength of living animal cells using SCFS, emphasizing cantilever calibration, cell immobilization, substrate preparation, contact time, retraction velocity, and data interpretation. Together, these studies established SCFS as a reproducible approach for measuring cellular adhesion beyond population-averaged assays. As an example of its biological relevance, Lopes et al. (2023) showed that fibrinogen increases both the strength and work of adhesion between erythrocytes, highlighting the potential of SCFS to identify biophysical markers associated with cell-cell interactions.

Viljoen et al. (2021) further consolidated AFM-based force spectroscopy as a versatile method for investigating the strength and dynamics of cell adhesion across different biological scales, from whole-cell interactions to single-molecule events. Their work emphasized that AFM-FS can quantify cellular adhesion, map cell-surface receptors, and evaluate dynamic mechanical and adhesive properties. This perspective is particularly relevant for biological interfaces because adhesion is not uniformly distributed across the cell surface; rather, it depends on receptor organization, cytoskeletal coupling, membrane mechanics, local biochemical composition, and the mechanical history of probe-sample contact. Therefore, AFM-derived adhesion curves should be interpreted not only as force readouts, but as local signatures of cellular organization and interfacial mechanics.

This dependence on membrane mechanics and biochemical composition is illustrated by studies of erythrocytes and cell–extracellular matrix interactions. Amorim et al. (2022) reported differences in the adhesion forces of normal and anemic erythrocytes and associated these changes with membrane structural and compositional alterations, including modifications in surface electrostatic distribution. Because the measurements were performed under low relative humidity using small-radius conical tips, capillary contributions were reduced, supporting the interpretation that long-range interactions, including electrostatic and van der Waals forces, contributed significantly to the measured adhesive response. Complementarily, Holuigue et al. (2023) used native extracellular matrix-modified AFM probes to investigate cell–microenvironment interactions and showed that cell adhesion strength is modulated by tissue-specific extracellular matrix composition and biomechanical heterogeneity through integrin-mediated mechanotransduction. Together, these examples show that AFM-based adhesion measurements can capture both intrinsic membrane alterations and microenvironment-dependent recognition processes.

In cancer research, AFM has been used to relate tumor cell morphology, nanomechanics, adhesion, and molecular interactions to malignant behavior. Deng et al. (2018) reviewed AFM applications in cancer research and highlighted that nanoscale changes in cell mechanics and adhesion are relevant to tumor progression, invasion, and metastasis. As an experimental example, Puech and colleagues (Puech et al. 2006) developed an AFM-based approach with an extended pulling range to quantify cell–cell adhesion forces between WM115 melanoma cells and human umbilical vein endothelial cells, showing that long-range unbinding events and cellular deformation must be considered when interpreting tumor cell adhesion. More recently, Wei et al. (2024) used SCFS with ConA-functionalized cantilevers to investigate interactions between gastric tumor cells and HMrSV5 mesothelial cells under fluid-flow conditions. They observed a significant reduction in cell adhesion after fluid culture and associated this response with changes in apical E-cadherin, suggesting that flow-induced biomechanical alterations can modulate adhesive behavior and favor migratory phenotypes relevant to tumor progression (Van Roy 2014; Wei et al. 2024).

The relationship between cellular mechanics, adhesion, and malignant behavior also extends to hematological tumors. Korayem and Mehrabani (2024) investigated chronic lymphocytic leukemia (CLL) cells using AFM-FS and reported that fresh cells exhibited higher adhesion strength and work of adhesion than fixed cells, indicating that membrane structural integrity strongly influences the adhesive response. These findings are relevant because the interaction of leukemic cells with bone marrow and lymphoid microenvironments is closely associated with cell retention, survival, and disease progression. Similarly, Lima et al. (2019) reported that acute myeloblastic leukemia (AML) cells showed greater resistance to detachment than healthy eosinophils, reinforcing the importance of adhesive and mechanobiological properties in leukemic cell–microenvironment interactions.

Functionalized AFM approaches have been particularly useful for investigating receptor-mediated mechanisms in cell-biomaterial adhesion. Harjumäki et al. (2020) applied AFM force spectroscopy to evaluate the role of integrins and their activation state in cell-biomaterial interactions. Their results showed that integrin expression and activation influence cell adhesion to biomaterial surfaces, demonstrating that AFM-FS can help distinguish receptor-mediated adhesion from global nonspecific adhesive responses. This distinction is especially relevant for biomaterial development, where biological performance depends not only on surface chemistry or topography, but also on how cells recognize and mechanically engage specific molecular ligands. In this context, Álvarez-López et al. (2023) used SCFS to evaluate adhesion forces between cells and peptide-decorated functionalized materials, proposing the technique as a strategy to identify peptide motifs that favor cell-biomaterial interactions. Together, these studies illustrate how AFM-based adhesion measurements can guide the design of bioactive surfaces by linking molecular functionalization, cellular recognition, and mechanical adhesive response.

In summary, studies involving cells and cell-biomaterial interfaces show that AFM-based adhesion measurements can quantify whole-cell adhesion, receptor-mediated recognition, and adhesive responses to engineered surfaces. These applications are relevant for understanding cell-matrix communication, tumor cell interactions with the endothelium, tissue engineering, implant development, and the design of biomaterials intended to regulate cell adhesion. Importantly, they also demonstrate that adhesion curves can capture both global interfacial properties and specific molecular contributions, depending on the probe configuration, surface functionalization, and biological scale of the experiment.

AFM-based adhesion measurements can also provide relevant information in tissue-level systems, where the adhesive response reflects not only cellular properties but also extracellular matrix composition, hydration, ionic environment, and hierarchical organization. Rojas et al. (2014) used AFM-based colloidal force spectroscopy with aggrecan-functionalized probes to investigate adhesive interactions in articular cartilage. They observed that adhesion strength and adhesion energy increased with ionic strength and Ca^2+^ concentration, and that enzymatic degradation of the collagen network enhanced aggrecan-collagen adhesion. These findings indicate that molecular adhesion within the cartilage extracellular matrix contributes to energy dissipation and to the mechanical behavior of the tissue.

AFM can also map biophysical alterations in complex tissues affected by pathological conditions, providing local information on adhesion, stiffness, morphology, and surface organization under minimally disruptive preparation conditions (Cho et al. 2023; Krieg et al. 2018). In human corneas affected by bullous keratopathy, Dias Rates et al. (2024) reported significant changes in endothelial and epithelial adhesion forces compared with healthy tissues. The pathological endothelium exhibited reduced adhesion, which was associated with loss of membrane components and altered ionic distribution, whereas the epithelium showed increased adhesion consistent with surface degradation and biochemical remodeling. Because the measurements were performed with non-functionalized small-radius conical probes under low relative humidity, capillary contributions were reduced, supporting the interpretation that long-range interactions, including van der Waals and electrostatic contributions, played an important role in the measured adhesive response.

In hippocampal tissues from rats with type 1 and type 2 diabetes mellitus, AFM-based analysis revealed reduced adhesion forces in hyperglycemic groups compared with controls, with a more pronounced decrease in the type 2 diabetes group (Do Nascimento Amorim et al. 2024). This reduction was accompanied by tissue softening, reflected by lower Young’s modulus values, while local spatial correlations suggested that regions with higher stiffness could also display higher adhesion. These findings were interpreted as consequences of microstructural and biochemical alterations associated with diabetes, including extracellular matrix remodeling, protein aggregation, oxidative stress, and changes in tissue surface organization. Because measurements were performed under comparable temperature and humidity conditions using small-radius probes and PeakForce QNM mode, the observed adhesion differences were discussed in terms of long-range interactions, including electrostatic and van der Waals contributions. Possible probe electrification associated with high-frequency tip-sample interactions in air may also contribute to the measured adhesive response and should be considered when interpreting adhesion data obtained under these experimental conditions.

### Parasitic, bacterial, and fungal adhesion: from single-molecule recognition to multicellular biofilms

Parasitic, bacterial, and fungal adhesion represents a critical early step in colonization, host–pathogen interaction, and biofilm formation. AFM-based force spectroscopy has contributed significantly to this field by enabling adhesion forces to be quantified from the single-molecule to the single-cell level, shifting the analysis of microbial and pathogen surfaces from static structural description to dynamic adhesive behavior (Beaussart et al. 2012; Dufrêne 2015; Li et al. 2011). In this context, single-molecule force spectroscopy and single-cell force spectroscopy provide complementary perspectives: the former probes individual adhesins or defined molecular interactions, whereas the latter quantifies the collective adhesive response of whole cells (Beaussart and El-Kirat-Chatel 2019; Mathelié-Guinlet et al. 2019). Together, these approaches show that adhesion depends strongly on the organization of the cell wall or host-cell membrane, surface proteins, polysaccharides, capsules, pili, fimbriae, and extracellular polymeric substances (EPS), connecting nanoscale architecture to functional attachment mechanisms.

At the nanoscale, pathogen-induced changes in adhesion can be illustrated by *Plasmodium falciparum*-infected erythrocytes (Carvalho et al. 2013; Li et al. 2011; Scholz et al. 2025). This system is biophysically relevant because parasite infection remodels the erythrocyte membrane, altering its ultrastructure, surface chemistry, deformability, and adhesive phenotype, thereby contributing to microvascular sequestration associated with severe malaria. Early AFM and surface potential spectroscopy studies revealed parasite-induced membrane protrusions known as knobs, providing nanoscale information on structural and electrostatic changes in unfixed infected erythrocytes (Aikawa et al. 1996). Subsequent AFM analyses combined with optical microscopy correlated infection stage with the number and size of knobs, showing that AFM can quantify parasite-induced membrane remodeling at the single-cell level (Aikawa et al. 1996; Nagao et al. 2000).

Beyond topographic imaging, AFM-based force spectroscopy can probe molecular recognition events at the surface of *P. falciparum*-infected erythrocytes. Using heparin-functionalized AFM tips, Valle-Delgado et al. (2013) demonstrated specific binding to infected red blood cells, reporting adhesion forces in the range of 28–46 pN depending on the loading rate, with binding events preferentially detected in late-stage parasite forms. These results show that functionalized probes can distinguish infection-related molecular recognition from global surface remodeling. Because cytoadhesion in malaria depends on receptor-ligand binding, membrane deformability, contact mechanics, and hydrodynamic conditions, AFM-derived force data should be interpreted together with the structural and mechanical state of the infected cell (Helms et al. 2016; Perez-Guaita et al. 2018). Complementary nanoscale chemical methods, such as multispectral AFM-infrared nanoimaging, can further support this interpretation by mapping biochemical remodeling during infection, although they do not directly measure adhesion forces (Helms et al. 2016; Perez-Guaita et al. 2018).

Bacterial models further demonstrate that specific surface appendages and adhesins can dominate adhesion responses beyond global physicochemical properties such as charge, hydrophobicity, or roughness. Using SCFS, Sullan et al. (2014) showed that adhesion of *Lactobacillus rhamnosus* GG is strongly influenced by pili-mediated interactions, which shape both the magnitude and rupture profile of the measured forces. In host-associated bacterial adhesion, multiparametric AFM imaging combined with bacterial probes has enabled spatial mapping of interaction forces between *Staphylococcus aureus* and human skin corneocytes (Formosa-Dague et al. 2016). This approach revealed that *S. aureus* adhesion to corneocytes is regulated by the host skin environment, with natural moisturizing factor levels modulating adhesion strength in atopic dermatitis (Feuillie et al. 2018). At the molecular level, Da Costa et al. (2022) showed that fibronectin-binding protein B (FnBPB) binds to human loricrin, mediating weak and ultra-strong binding events reaching approximately 2 nN under mechanical stress. Together, these studies show how AFM can connect microbial adhesion signatures with pili, adhesins, host ligands, and disease-relevant surface conditions.

AFM-based adhesion measurements have also been used to investigate bacterial attachment to clinically relevant biomaterial surfaces. Alam and Balani (2017) used SCFS to compare the adhesion of *S. aureus* to stainless steel, Ti-6Al-4 V, and ultra-high-molecular-weight polyethylene (UHMWPE). They reported higher adhesion forces on metallic substrates than on UHMWPE, which they associated with differences in surface roughness and surface energy. These properties can increase the number of effective contact points between the bacterium and the substrate, favoring complex retraction profiles with multiple rupture events. This example illustrates how AFM can connect nanoscale bacterial adhesion signatures with material-dependent surface properties relevant to implant colonization and infection risk.

Microbial adhesion is also strongly influenced by contact time, molecular dynamics, and force-dependent binding mechanisms. Aguayo et al. (2015) showed that the maximum adhesion force of living *S. aureus* on machined titanium surfaces increased from approximately 0.27 nN to 9.15 nN as contact time increased to 60 s. This time-dependent strengthening was associated with the engagement and unfolding of surface adhesins, including FnBPA, with contour lengths of approximately 314 nm estimated using the worm-like chain model. In *Escherichia coli*, Yakovenko et al. (2008) demonstrated that the FimH adhesin forms catch bonds, in which mechanical tension induces an allosteric transition to a high-affinity state, increasing rupture forces from approximately 20–40 pN to 120–180 pN. These examples show that microbial adhesion curves can encode not only static surface affinity, but also dynamic bond strengthening, adhesin unfolding, and force-regulated molecular recognition.

Environmental conditions and surface topography further modulate microbial adhesion, particularly in biofilm-forming systems. Kannan and Gautam (2015) showed that adhesion forces in *Pseudomonas aeruginosa* biofilms varied with temperature and substrate roughness, with stronger adhesion observed under conditions that favored metabolic activity and extracellular polymeric substance production. The incorporation of divalent ions such as Ca^2+^ and Mg^2+^ can further stabilize the biofilm matrix by promoting polymer bridging and cohesive interactions within extracellular polymeric substances. Although biofilms introduce an additional level of complexity due to their heterogeneous multicellular organization, AFM-based mapping and force spectroscopy provide a way to investigate how extracellular polymers, surface roughness, and cohesive forces contribute to biofilm stability, persistence, and dispersal on technologically and clinically relevant surfaces (James et al. 2017).

Fungal pathogens further illustrate how AFM-FS can connect cell-wall architecture, molecular recognition, and adhesion dynamics. Awassa et al. (2024) investigated nanoscale adhesion forces involving *Candida albicans* and zinc-based layered double hydroxides using single-cell and single-particle force spectroscopy. They reported ion-dependent adhesive responses, with Zn^2+^ showing a specific affinity for Als proteins in the fungal cell wall and producing protein-unfolding signatures with rupture lengths reaching hundreds of nanometers. These findings indicate that AFM force curves can detect not only adhesion magnitude, but also molecular unfolding events associated with fungal surface proteins.

The adhesive behavior of *C. albicans* is also strongly influenced by morphology and interspecies interactions. Jackson et al. (2014) showed that the transition from yeast to hyphal forms affects biofilm development on denture acrylic resin surfaces, supporting the relevance of cell shape in fungal adhesion. Using colloidal force spectroscopy with PMMA microspheres, adhesion to acrylic surfaces was shown to be cell-shape dependent, with stronger attachment associated with germ-tube or hyphal forms than with yeast-phase cells. In inter-kingdom interactions, Ovchinnikova et al. (2012) demonstrated that *Staphylococcus aureus* adhesion varies along the length of *C. albicans* hyphae, with stronger interactions in the apical and middle regions than in basal regions or budding yeasts. Together, these fungal and inter-kingdom examples show that AFM-based adhesion measurements can reveal how morphology, cell-wall proteins, substrate chemistry, and microbial partnerships shape colonization and biofilm-associated adhesion.

### Viral particles and glycan-mediated adhesion

Viral particles represent a particularly important class of biological systems for AFM-based adhesion studies because infection begins with the physical attachment of virions to host-cell surfaces. This initial contact is often multivalent and mechanically regulated, involving viral proteins, envelope glycoproteins, glycans, glycosaminoglycans, lipid envelopes, and host receptors. Kuznetsov and McPherson (2011) reviewed the use of AFM for imaging viruses and virus-infected cells, highlighting that AFM can examine viral particles at nanometer resolution under conditions compatible with biological samples. Although early AFM studies in virology focused mainly on morphology, capsid organization, and structural stability, they established the experimental basis for later force spectroscopy approaches aimed at measuring virus-host interactions, receptor binding, and mechanically mediated viral adhesion.

More recent reviews have expanded this perspective by positioning AFM as a tool for physical virology and single-particle analysis. Rousso and Deshpande (2022) reviewed applications of AFM in HIV-1 research, emphasizing its ability to image individual virions, viral components, and infected cells while probing mechanical properties under biologically relevant conditions. Ray and colleagues (Ray et al. 2025) further highlighted the contribution of AFM to the study of viral assembly, budding, host-ligand binding, and single-virus-level interactions. In the context of adhesion, these contributions are important because they show that AFM can move beyond static structural characterization and provide access to the mechanical and molecular events that support viral attachment, receptor engagement, and entry.

Furthermore, supported lipid bilayers (SLBs) serve as valuable biomimetic platforms to investigate viral adhesion to specific membrane microdomains. Bally et al. (2012) demonstrated that norovirus virus-like particles (VLPs) selectively recognize and bind to galactosyl ceramide glycolipids segregated within microdomains of planar SLBs, underscoring the power of combining AFM characterization with artificial membrane models to resolve domain-specific viral interactions. Beyond biomedical host-cell interactions, AFM is also gaining recognition in food safety and environmental virology to gather biophysical parameters of foodborne viruses (Dos Santos Natividade et al. 2025). In these contexts, AFM has been applied to quantify viral adhesion to food-processing surfaces and to elucidate the ionic-dependent deposition kinetics of enteric viruses in aquatic environments (Dos Santos Natividade et al. 2025).

Single-virus force spectroscopy has provided direct evidence that AFM can resolve specific viral attachment mechanisms. Delguste et al. (2021) used single-virus force spectroscopy to discriminate the intrinsic roles of two murid herpesvirus 4 glycoproteins, gp70 and gH/gL, during attachment to cell-surface glycosaminoglycans. By comparing force signatures associated with different viral glycoproteins, the authors showed that gH/gL was the main contributor to glycosaminoglycan binding, generating more frequent and more stable interactions than gp70. They also demonstrated that Fab antibody fragments targeting gH/gL or gp70 reduced virion attachment to cell surfaces. This study is particularly relevant because it shows that AFM-derived adhesion curves can identify which viral surface components dominate the initial adhesive step of infection and can evaluate how molecular blocking strategies interfere with virus–cell attachment.

AFM-FS has also been used to investigate molecular interactions involving SARS-CoV-2. Yang et al. (2020) applied single-molecule force spectroscopy to quantify interactions between the SARS-CoV-2 spike glycoprotein and the ACE2 receptor, showing that AFM can resolve molecular binding events underlying viral attachment and provide kinetic and thermodynamic information on spike–receptor interactions. Simpson et al. (2023) further examined interactions between the SARS-CoV-2 S1 protein and C-type lectin receptors using force-distance curve-based AFM combined with biolayer interferometry. By comparing the receptor-binding domain, deglycosylated S1, and a non-glycosylated S1 variant, the authors demonstrated that glycosylation contributes to these interactions. Together, these studies illustrate how functionalized AFM approaches can dissect molecular determinants of viral adhesion to host receptors or receptor-like molecules, linking force signatures to receptor engagement, glycan recognition, and binding specificity.

AFM-based adhesion mapping has also contributed to the nanoscale characterization of SARS-CoV-2 particles. Cardoso-Lima et al. (2021) reported adhesion-force maps showing a heterogeneous viral surface with spike-associated features organized in triangular arrangements of approximately 10.9 nm. These adhesion signatures, combined with topographic and nanomechanical information, contributed to the identification of surface domains associated with viral protein organization. After controlled adsorption on glass substrates, the authors also reported nanoscale features attributed to internal nucleocapsid arrangements, including hexameric and tetrahedral assemblies. Although these measurements should be interpreted in combination with complementary structural and biochemical evidence, they illustrate how AFM adhesion mapping can provide indirect information on viral surface organization, local charge distribution, and multivalent interaction sites relevant to viral attachment.

In Mayaro virus particles, França et al. (2024) reported nanoscale surface features marked by triangular arrangements and local fluctuations in adhesion forces. These adhesion variations were interpreted as reflecting heterogeneity in surface charge distribution and the spatial organization of E1/E2 envelope protein domains. The force curves also exhibited a tether-like retraction behavior, characterized by an initial abrupt detachment event followed by long-range plateau-shaped interactions. Such profiles suggest that, after the first rupture event, molecular or envelope-associated structures may remain transiently connected to the AFM tip and be mechanically extended before final detachment. This example illustrates how AFM adhesion signatures can provide information not only on the magnitude of viral adhesion, but also on local surface heterogeneity, envelope organization, and mechanically mediated detachment processes.

A particularly illustrative example of how force-curve shape can reveal viral surface organization was provided by Ogharandukun et al. (2020), who investigated self-adhesion and aggregation of N-glycan sugars using virus glycan shields. In this study, force spectroscopy measurements between pseudotyped viruses bearing complex N-glycan shields revealed distinct adhesion profiles depending on the mechanical sampling conditions. Slime-like adhesion was observed at higher ramp rates, whereas Velcro-like adhesion appeared at lower ramp rates. The authors interpreted these profiles as a consequence of rate-dependent access to different regions of the glycan shield: faster probing compressed the viral surface and favored interactions involving external sialic acid-containing glycans, whereas slower probing allowed deeper penetration into the glycan layer and access to mannose-rich core interactions. The disappearance or alteration of these signatures after enzymatic cleavage of sialic acid or mannose further supported their molecular origin. This study is especially relevant because it demonstrates that AFM retraction profiles can report not only adhesion magnitude, but also glycan accessibility, molecular architecture, multivalency, and dissipative detachment at viral interfaces.

Taken together, these viral studies show that AFM-FS can connect adhesion signatures with glycoprotein accessibility, glycan architecture, multivalent binding, receptor engagement, and virion mechanics. This is particularly relevant for enveloped viruses, in which glycoprotein distribution, glycan masking, membrane compliance, and local charge heterogeneity collectively influence the probability, specificity, and mechanical stability of host-cell attachment. Importantly, viral adhesion curves should not be interpreted only by their maximum pull-off force. Multiple rupture events, tether-like profiles, slime-like adhesion, and Velcro-like responses can provide complementary information on how viral surfaces are organized and mechanically sampled during probe retraction. In the broader context of this review, viral particles demonstrate how AFM adhesion measurements can act as structural, molecular, and mechanical fingerprints of nanoscale biological interfaces.

### Comparative overview of AFM-based adhesion studies in biological systems

The studies discussed above demonstrate the diversity of AFM-based strategies used to investigate adhesion in biological systems. These approaches include single-cell force spectroscopy, single-molecule force spectroscopy, functionalized AFM probes, bacterial probes, colloidal probes, force mapping, and single-virus force spectroscopy. Together, they show that AFM can probe adhesion across multiple biological scales, from defined receptor–ligand interactions to whole-cell, microbial, viral, tissue, and biomaterial interfaces.

Table 1 summarizes representative peer-reviewed studies in which AFM-derived adhesion parameters, retraction-curve signatures, or spatial adhesion maps were connected to biological mechanisms. Rather than providing an exhaustive catalog, the table highlights examples that illustrate how adhesion curves can reveal molecular recognition, membrane or surface remodeling, multivalent attachment, tether formation, dissipative detachment, and material-dependent biological responses.

**Table 1:** Representative peer-reviewed studies illustrating how AFM-based adhesion measurements connect force-curve readouts with biological mechanisms. The table highlights selected examples across cellular, tissue, microbial, fungal, viral, and biomaterial-related systems. Emphasis is placed on the AFM strategy used, the adhesion-related readout or curve signature, and the biological insight obtained from the measurement. The table is not intended as an exhaustive catalog, but as a comparative framework showing how AFM-derived adhesion signatures can reveal molecular recognition, surface remodeling, multivalent attachment, tether formation, dissipative behavior, and material-dependent biological responses

Biological system/application	AFM strategy	Adhesion signature/readout	Biological insight	Representative studies
Living cells and cell-cell or cell-substrate adhesion	Single-cell force spectroscopy	Adhesion force, work of adhesion, detachment dynamics, and controlled contact-time-dependent adhesion	Established SCFS as a quantitative strategy to measure adhesion of living cells under controlled contact force, contact time, and retraction conditions, enabling cellular adhesion to be analyzed beyond population-averaged assays	(Friedrichs et al. 2013; Helenius et al. 2008; Viljoen et al. 2021)
Erythrocytes and pathological membrane alterations	AFM force spectroscopy and SCFS	Changes in adhesion force and work of adhesion associated with membrane properties	Demonstrated that AFM-derived adhesion parameters can reveal altered membrane organization, cell-cell interactions, and biophysical changes in normal and pathological erythrocytes	(Amorim et al. 2022; Lopes et al. 2023)
Cancer cell adhesion and tumor-related interactions	AFM-based cell-cell adhesion assays and SCFS	Long-range detachment, cell deformation, adhesion force, and altered adhesive response under flow	Showed that AFM can connect tumor cell adhesion with deformation, endothelial or mesothelial interactions, E-cadherin-associated responses, and biomechanical features relevant to invasion and metastasis	(Deng et al. 2018; Puech et al. 2006; Wei et al. 2024)
Hematological tumor cells	AFM force spectroscopy	Adhesion strength, work of adhesion, and resistance to detachment	Indicated that leukemic cell adhesion is influenced by membrane integrity and mechanobiological properties, supporting the relevance of adhesion in tumor cell-microenvironment interactions	(Korayem and Mehrabani 2024; Lima et al. 2019)
Cell–biomaterial interfaces	Functionalized AFM probes and SCFS	Integrin-mediated adhesion, receptor-dependent binding, and cell response to functionalized surfaces	Demonstrated that AFM-FS can distinguish receptor-mediated adhesion from global nonspecific adhesion and guide the design of bioactive biomaterial surfaces	(Álvarez-López et al. 2023; Harjumäki et al. 2020)
Tissue extracellular matrix and cartilage	Colloidal force spectroscopy with functionalized probes	Adhesion strength and adhesion energy between matrix macromolecules	Showed that molecular adhesion within cartilage ECM depends on ionic strength, Ca^2+^ concentration, and collagen network integrity, contributing to tissue energy dissipation and mechanical behavior	(Rojas et al. 2014)
Pathological tissues	AFM force spectroscopy mapping and nanomechanical profiling	Spatial variations in adhesion force, stiffness, and surface organization	Revealed that tissue pathology can alter local adhesion and mechanics, linking AFM readouts to membrane degradation, ionic distribution, extracellular matrix remodeling, oxidative stress, and surface heterogeneity	(Dias Rates et al. 2024; Do Nascimento Amorim et al. 2024)
*Plasmodium falciparum*-infected erythrocytes	AFM imaging, surface potential spectroscopy, and heparin-functionalized SMFS	Knob morphology, surface remodeling, and specific heparin-binding forces	Demonstrated that AFM can detect parasite-induced membrane remodeling and that functionalized tips can identify infection-stage-dependent molecular recognition events at infected erythrocyte surfaces	(Aikawa et al. 1996; Nagao et al. 2000; Valle-Delgado et al. 2013)
Pili- and adhesin-mediated bacterial adhesion	SCFS and SMFS	Multiple rupture events, adhesin unfolding, contact-time-dependent strengthening, and force-regulated binding	Showed that bacterial adhesion is governed by specific appendages and adhesins, including pili, FnBPA/FnBPB-related interactions, and FimH catch bonds, rather than by global surface properties alone	(Aguayo et al. 2015; Da Costa et al. 2022; Sullan et al. 2014; Yakovenko et al. 2008)
Bacterial adhesion to host surfaces	Multiparametric AFM imaging and bacterial probes	Spatially resolved interaction forces between bacteria and host cells or tissues	Revealed that bacterial adhesion to host surfaces depends on local tissue composition, host ligands, and disease-associated surface conditions, such as natural moisturizing factor levels in atopic dermatitis	(Da Costa et al. 2022; Feuillie et al. 2018; Formosa-Dague et al. 2016)
Bacterial adhesion to biomaterials	SCFS using bacterial probes	Substrate-dependent adhesion force and multiple rupture events	Demonstrated that material properties such as surface roughness and surface energy modulate bacterial attachment, favoring multiple contact points and complex detachment profiles relevant to implant colonization	(Alam and Balani 2017)
Biofilms and extracellular polymeric substances	AFM force spectroscopy and force mapping	Adhesion forces, cohesive interactions, EPS-mediated adhesion, and environmental modulation	Showed that biofilm adhesion is influenced by temperature, substrate roughness, divalent ions, EPS production, and matrix cohesion, linking AFM readouts to biofilm stability and persistence	(James et al. 2017; Kannan and Gautam 2015)
Fungal adhesion and inter-kingdom interactions	SCFS, single-particle force spectroscopy, and colloidal force spectroscopy	Ion-dependent adhesion, protein unfolding signatures, morphology-dependent attachment, and spatially heterogeneous adhesion	Demonstrated that fungal adhesion depends on cell-wall proteins, ion-specific interactions, yeast-to-hypha morphology, substrate chemistry, and bacterial–fungal interactions	(Awassa et al. 2024; Jackson et al. 2014; Ovchinnikova et al. 2012)
Viral glycoprotein-mediated attachment	Single-virus force spectroscopy and functionalized AFM approaches	Specific rupture forces, stable binding events, and inhibition-sensitive adhesion	Demonstrated that AFM-derived adhesion curves can discriminate viral surface components involved in attachment and evaluate how antibodies or molecular blocking strategies interfere with virus–cell binding	(Delguste et al. 2021; Simpson et al. 2023; Yang et al. 2020)
Viral surface organization and tether-like detachment	AFM force spectroscopy mapping	Local adhesion heterogeneity, multiple rupture events, and tether-like retraction profiles	Showed that viral adhesion maps and retraction profiles can provide information on surface organization, envelope protein distribution, local charge heterogeneity, and mechanically mediated detachment processes	(Cardoso-Lima et al. 2021; Silva França et al. 2024)
Glycan-mediated viral adhesion	Force spectroscopy between glycan-covered viral particles	Slime-like and Velcro-like retraction profiles, rate-dependent adhesion, and multivalent detachment	Demonstrated that the shape of AFM retraction curves can report glycan accessibility, molecular architecture, multivalent contacts, and dissipative behavior in glycan-rich viral interfaces	(Ogharandukun et al. 2020)

As a whole, the evidence presented in Table 1 demonstrates that AFM-based adhesion measurements have progressed from simple force quantification to a family of approaches capable of resolving global interfacial adhesion, receptor-ligand recognition, cell-substrate interactions, microbial adhesins, pathogen-induced surface remodeling, virus-receptor binding, glycan-mediated multivalence, and biomaterial-dependent responses. Across these systems, the most revealing studies interpret adhesion curves as multidimensional signatures rather than single pull-off force values. Adhesion force, work of adhesion, interaction length, rupture-event distribution, tether formation, dissipative profiles, and spatial adhesion heterogeneity can all provide valuable insights into molecular organization, surface architecture, mechanical coupling, and biological function. This comparative perspective reinforces the central argument of this review: AFM retraction curves should be interpreted as context-dependent biophysical fingerprints that connect nanoscale force measurements with biological mechanisms.

## Current limitations and future perspectives

Despite the broad potential of AFM-based force spectroscopy for studying adhesion in biological systems, several methodological and interpretative limitations still restrict direct comparison between studies. Adhesion measurements are highly sensitive to instrumental parameters, including cantilever calibration, probe geometry, tip radius, probe chemistry, applied force, contact time, retraction velocity, and sampling rate (Butt et al. 2005; Cappella and Dietler 1999; Hinterdorfer and Dufrêne 2006). Environmental conditions, such as buffer composition, ionic strength, pH, temperature, and hydration state, further modulate both nonspecific and specific interactions (Butt et al. 2005; Israelachvili 2011). In biological samples, additional variability arises from cell viability, passage number, membrane integrity, extracellular matrix adsorption, microbial growth phase, biofilm maturity, viral particle preservation, sample immobilization, and intrinsic heterogeneity (Morris et al 1999; Dufrêne 2008; Dufrêne et al. 2013; Viljoen et al. 2021). Therefore, detailed reporting of experimental conditions is essential for reproducibility and for meaningful comparison across datasets obtained from cells, tissues, microorganisms, biomaterials, and viral particles (Butt et al. 2005; Friedrichs et al. 2013; Viljoen et al. 2021).

A significant limitation in the literature is that adhesion data are often reported mainly as maximum pull-off force values, with limited information about the complete retraction profile or the experimental context in which the curves were acquired. Although *F*_*adh*_ is useful and experimentally accessible, it does not fully describe biological adhesion, especially at soft, hydrated, and polymer-rich interfaces. Reporting only average adhesion forces can obscure relevant differences in adhesion work, interaction length, rupture-event distribution, tether formation, loading-rate dependence, and dissipative behavior. Future studies should therefore report complementary curve descriptors and their statistical distributions, rather than relying exclusively on mean pull-off forces. This practice would improve comparisons between cells, tissues, microorganisms, viral particles, biomaterials, and pathogen-modified interfaces, where adhesion mechanisms differ substantially in molecular origin, spatial organization, and mechanical response.

Another important challenge concerns the distinction between nonspecific adhesion and specific molecular recognition. Nonspecific interactions, including electrostatic, van der Waals, hydrophobic, hydration, and steric contributions, form the physical background of most biological adhesion measurements and can strongly influence probe–sample contact even when molecular specificity is not intended (Müller et al. 2021). Functionalized probes increase molecular selectivity by allowing defined ligands, antibodies, peptides, glycans, receptors, or extracellular matrix components to be immobilized at the AFM tip. However, this strategy also introduces additional sources of uncertainty. Molecular immobilization can restrict conformational and orientational freedom, alter binding-site accessibility, and favor multiple simultaneous interactions, making quantitative interpretation of rupture forces more complex (Müller et al. 2021). Therefore, specificity should not be inferred from force peaks alone. Future studies should combine functionalized probes with rigorous controls, including blocking assays, competition experiments, enzymatic treatments, receptor inhibition, genetic perturbation when applicable, and comparison with non-functionalized or control-functionalized probes.

Furthermore, a crucial analytical strategy to validate specific single-molecule interactions is the quantitative fitting of retraction curves using theoretical polymer elasticity models, most notably the Worm-like-Chain (WLC) and Freely-Jointed Chain (FJC) models (Hinterdorfer and Dufrêne 2006; Marko and Siggia 1995). When functionalized probes employ flexible polymer linkers (e.g., polyethylene glycol, PEG) or when mechanical protein unfolding occurs, such as the sequential unfolding of bacterial adhesins like fibronectin-binding protein A (FnBPA), which displays characteristic molecular contour lengths of ~ 314 nm (Yakovenko et al. 2008), fitting the nonlinear force-extension region yields intrinsic physical parameters including contour length (${L}_{c}$) and persistence length (p). Matching these extracted parameters to the known molecular dimensions of the polymer linker or protein construct confirms that a rupture peak arises from the elastic stretching and unbinding of a discrete, single-molecule tether rather than nonspecific tip-sample sticking (Hinterdorfer and Dufrêne 2006; Marko and Siggia 1995).

Biological heterogeneity represents both a limitation and an opportunity in AFM-based adhesion studies. Living cells, microbial populations, biofilms, biomaterial surfaces, tissues, and viral preparations are rarely homogeneous. Adhesion can vary across different cellular regions, between individual cells, along microbial growth phases, within biofilm domains, among viral particles, or across chemically and topographically distinct regions of biomaterial surfaces (Fang et al. 2000; Müller and Dufrêne 2008; Silva França et al. 2024). This variability complicates statistical analysis and cross-study comparison, but it can also provide biologically meaningful information about spatial organization, phenotypic diversity, surface remodeling, and local mechanical or biochemical states. Force-volume mapping, spatial adhesion maps, SCFS, and SMFS can help capture this variability, provided that studies include a sufficient number of curves, transparent exclusion criteria, appropriate statistical treatment, and reporting of full distributions or subpopulations rather than relying only on average values.

Although AFM is widely used for the nanomechanical characterization of biological tissues (Cho et al. 2023; Krieg et al. 2018; Okajima and Kuribayashi-Shigetomi 2023; Zhang et al. 2025), studies that directly report and interpret adhesion forces in tissues remain relatively scarce compared with analyses of stiffness, elasticity, and viscoelasticity. In many tissue studies, adhesion is acquired as a complementary parameter during force mapping, but its biological meaning is not always explored in depth. Conversely, approaches based on AFM tips functionalized with extracellular matrix components are frequently used to investigate specific cell-matrix or matrix-matrix interactions (Helenius et al. 2008; Lehenkari and Horton 1999; Li et al. 2003); however, these measurements probe molecularly defined binding events and are not directly comparable to nonspecific adhesion obtained with non-functionalized probes. Future studies on tissue adhesion should therefore clearly distinguish global tissue adhesive fingerprints from specific molecular recognition assays and should interpret adhesion together with local mechanics, extracellular matrix organization, hydration, and biochemical composition.

Throughput remains a significant bottleneck in biological AFM. Force spectroscopy provides rich nanoscale information, but it is relatively slow compared with optical, microfluidic, or ensemble-based adhesion assays, limiting the number of cells, microorganisms, viral particles, tissue regions, or biomaterial domains that can be analyzed in a single experiment. This limitation is particularly relevant for adhesion studies because biological heterogeneity often requires large datasets to distinguish meaningful subpopulations from experimental variability. Recent efforts in high-throughput cellular AFM, automation, and artificial intelligence aim to increase the number of measured cells and force curves while reducing operator-dependent variability (Thomas-Chemin et al. 2025). These developments may improve statistical robustness in adhesion studies, provided that increased acquisition speed is accompanied by rigorous calibration, curve-quality control, and transparent reporting of preprocessing and classification criteria.

Data analysis and curve classification represent another critical frontier in AFM-based adhesion studies. Biological force curves can contain no-rupture events, single rupture events, multiple rupture events, tether-like profiles, viscous tails, hydrodynamic artifacts, nonspecific sticking, or mixed signatures that combine several of these features. Manual classification of such profiles is time-consuming and can introduce user-dependent bias, especially when large datasets are required to capture biological heterogeneity. Machine learning and deep learning approaches are beginning to address this limitation. Waite et al. (2023) developed a few-shot deep learning strategy for classifying AFM force curves from single-molecule interaction experiments, including curves with and without rupture events. More broadly, Masud et al. (2024) reviewed how machine learning can improve AFM instrumentation and data analytics, including automated force-curve analysis and high-throughput processing. In the future, such approaches may be particularly useful for identifying adhesion signatures such as tether-like, Velcro-like, slime-like, and multipeak profiles, provided that training datasets are well annotated, experimentally validated, and interpreted within the appropriate biological and physical context.

Another promising direction is the integration of AFM force spectroscopy with complementary imaging and spectroscopic techniques (Dufrêne et al. 2017; Hinterdorfer and Dufrêne 2006). AFM can already combine topography, nanomechanics, adhesion, dissipation, and force mapping; however, biological interpretation often requires additional information on molecular composition, receptor distribution, cytoskeletal organization, biochemical state, or dynamic structural remodeling (Butt et al. 2005; Garcia and Herruzo 2012; Hinterdorfer and Dufrêne 2006; Krieg et al. 2018). Correlative approaches involving fluorescence microscopy, Raman spectroscopy, infrared nanospectroscopy, AFM-IR, KPFM, high-speed AFM, or electron microscopy may help connect adhesion signatures with molecular identity and biological function (Ando et al. 2024; Dufrêne et al. 2017; Gao et al. 2018; Prats‐Mateu and Gierlinger 2017; Quaroni et al. 2017; Rahimi et al. 2025; V. D. Dos Santos et al. 2023; Zhou et al. 2017). This integration is particularly relevant for complex interfaces such as infected cells, biofilms, tumor microenvironments, biomaterials, and enveloped viruses, where adhesion depends simultaneously on nanoscale architecture, biochemical composition, local charge distribution, hydration, and mechanical compliance (De Pablo and Schaap 2019; Krieg et al. 2018). In this context, multimodal workflows can help determine whether a given adhesion profile reflects specific molecular recognition, polymer-rich surface layers, membrane or envelope deformation, electrostatic heterogeneity, or dissipative detachment.

Standardization will be essential for the field to mature. Although AFM-based adhesion studies have generated valuable insights into biological interfaces, differences in cantilever selection, probe geometry, tip radius, probe functionalization, loading protocol, environmental conditions, data processing, and statistical reporting still limit cross-study comparison. Future experimental studies and reviews would benefit from community-oriented reporting guidelines for AFM-based adhesion measurements in biological systems. At minimum, such guidelines should include information on probe calibration and geometry, functionalization protocols, measurement conditions, force-curve acquisition parameters, preprocessing criteria, statistical treatment, and reporting of full parameter distributions.

A major driver of this standardization bottleneck lies in the data analysis workflow itself. Variations in preprocessing algorithms, such as baseline tilt correction, zero-force assignment, and contact-point determination, can introduce significant discrepancies in reported adhesion parameters across different software platforms (ranging from proprietary vendor software to open-source packages). Furthermore, cross-study comparability is hindered by the choice of fitting models; for instance, applying distinct kinetic models (e.g., Bell-Evans or Dudko-Hummer-Szabo) or varying viscoelastic fitting frameworks to identical retraction curves can yield markedly different kinetic off-rates (${k}_{off}^{0}$) and transition-state distances (Δx). Adopting transparent and reproducible processing workflows, preferably using well-documented software tools or open-source pipelines when available, and reporting key analysis parameters are therefore important steps toward harmonizing AFM force-distance curve analysis across laboratories. Establishing these reporting practices would improve reproducibility, reduce interpretative ambiguity, and facilitate comparisons across cells, tissues, microorganisms, biomaterials, and viral particles.

To translate these considerations into a practical framework, Table 2 summarizes a recommended reporting checklist for AFM-based adhesion studies in biological systems. The checklist is not intended to impose a single experimental protocol, because adhesion measurements must be adapted to the biological system, probe configuration, and scientific question. Instead, it identifies minimum experimental, analytical, and statistical information that should be reported to improve reproducibility, reduce interpretative ambiguity, and facilitate comparisons across studies. By encouraging the reporting of probe characteristics, functionalization strategies, environmental conditions, force spectroscopy parameters, curve-processing criteria, adhesion descriptors, and statistical distributions, this checklist supports a more transparent and multidimensional interpretation of AFM-derived adhesion data.

**Table 2:** Recommended reporting checklist for AFM-based adhesion studies in biological systems. The checklist summarizes minimum experimental, analytical, and statistical information that should be reported to improve reproducibility, transparency, and cross-study comparison

Category	Recommended information to report	Why it matters
AFM probe and calibration	Cantilever model, nominal and calibrated spring constant, calibration method, deflection sensitivity, tip geometry, tip radius, and probe coating	Determines force accuracy, contact geometry, and comparability between experiments
Probe functionalization	Functionalization chemistry, ligand or molecule used, linker type, ligand density when available, passivation strategy, and control-functionalized probes	Defines molecular specificity and helps distinguish specific recognition from nonspecific adhesion
Sample preparation	Biological system, cell type or strain, passage number or growth phase, tissue region, viral preparation, immobilization method, substrate, and preservation conditions	Strongly influences surface structure, viability, mechanics, and adhesive response
Environmental conditions	Medium or buffer composition, ionic strength, pH, temperature, relative humidity, hydration state, or liquid environment	Modulates electrostatic, hydration, capillary, hydrophobic, and molecular recognition forces
Force spectroscopy protocol	Contact force or trigger force, contact time, approach velocity, retraction velocity, ramp size, sampling rate, number of force curves, and number of independent biological replicates	Controls bond formation, loading rate, rupture dynamics, tether formation, and statistical robustness
Controls for specificity	Non-functionalized probes, blocked probes, competition assays, enzymatic treatments, receptor inhibition, genetic perturbation when applicable, and negative controls	Prevents overinterpretation of force peaks as specific molecular binding events
Data preprocessing	Baseline correction, contact point determination, conversion of deflection to force, separation correction, drift correction, and criteria for curve exclusion	Ensures that adhesion parameters are extracted consistently and transparently
Adhesion parameters	Maximum adhesion force, work of adhesion, interaction length, rupture-event number, rupture-force distribution, loading-rate dependence, and curve classes	Captures adhesion as a multidimensional response rather than a single pull-off force
Curve classification	Criteria for identifying no-rupture, single pull-off, multiple rupture, sawtooth-like, tether-like, Velcro-like, slime-like, or mixed profiles	Enables mechanistic interpretation of retraction profiles and comparison between biological systems
Statistical reporting	Number of curves, number of cells or particles, number of independent samples, distribution plots, statistical tests, effect sizes, and replicate structure	Avoids overreliance on mean values and accounts for biological heterogeneity
Spatial analysis	Force-volume map size, pixel density, scan region, adhesion maps, stiffness maps, correlation with topography, and regional heterogeneity	Links local adhesion signatures to spatial organization and biological structure
Data transparency	Representative curves, full distributions, raw or processed data availability when possible, and analysis scripts or software settings	Improves reproducibility and enables reanalysis or comparison across studies

The adoption of such reporting practices would not eliminate the intrinsic complexity of biological adhesion, but it would make AFM-based measurements more reproducible, interpretable, and comparable across laboratories. Importantly, this checklist also reinforces the central concept of this review: adhesion should be reported as a multidimensional force-curve response that includes force magnitude, energy, interaction range, rupture dynamics, spatial heterogeneity, and biological context.

In the coming years, AFM-based adhesion analysis is likely to move toward more integrated, automated, and multimodal workflows. The combination of well-controlled force spectroscopy, spatial adhesion mapping, robust probe functionalization, high-throughput acquisition, machine learning-based curve classification, and correlative biochemical imaging could transform adhesion measurements into more reproducible and biologically interpretable datasets. However, these advances will only be fully effective if accompanied by rigorous experimental controls, transparent reporting, and careful interpretation of force-curve signatures within their biological and physical context. In this scenario, AFM will not only quantify how strongly biological systems adhere, but also reveal how molecular organization, surface architecture, mechanical compliance, and dynamic remodeling shape adhesion across biological scales.

## Conclusion

Adhesion is a central biophysical property of biological interfaces, governing processes that range from cell-cell and cell-matrix communication to microbial colonization, pathogen–host recognition, viral attachment, and the performance of biomaterials. AFM-based force spectroscopy provides a unique experimental approach to investigate these interactions at the nanoscale, allowing adhesive responses to be quantified under controlled conditions and, in many cases, in environments compatible with biological samples.

This review emphasizes that adhesion in biological systems should not be interpreted solely as a maximum pull-off force. Although *F*_*adh*_ remains an important and widely reported parameter, it represents only one component of a complex interfacial response. Work of adhesion, interaction length, rupture-event distribution, loading-rate dependence, spatial heterogeneity, and the overall shape of the retraction curve provide complementary information on the molecular, mechanical, and dissipative processes involved in detachment.

Across cells, tissues, biomaterials, microorganisms, pathogen-modified cells, and viral particles, AFM-FS has revealed that adhesion emerges from the coupling between nonspecific physicochemical interactions, specific molecular recognition, surface architecture, and mechanical compliance. Non-functionalized probes provide global adhesive fingerprints of biological interfaces, whereas functionalized probes, colloidal probes, SCFS, SMFS, and single-virus force spectroscopy enable selected molecular interactions, collective adhesive responses, and multiscale biological mechanisms to be investigated with high sensitivity.

The studies discussed in this review show that AFM-derived adhesion signatures can connect nanoscale force measurements with biological interpretation. Abrupt detachment events, multiple rupture peaks, sawtooth-like profiles, membrane or polymer tethering, slime-like adhesion, and Velcro-like patterns should not be viewed merely as experimental variations. Instead, when interpreted together with probe configuration, experimental controls, environmental conditions, and complementary structural or biochemical information, these signatures can reveal how biological interfaces are organized, how molecules are distributed or accessed, and how soft or complex surfaces respond to mechanical separation.

Future progress in this field will depend on improved standardization, rigorous experimental controls, robust statistical analysis, high-throughput acquisition, automated force-curve classification, and integration with complementary imaging and spectroscopy techniques. The adoption of transparent reporting practices, including detailed information on probe characteristics, functionalization, measurement conditions, curve processing, adhesion descriptors, and statistical distributions, will be essential to improve reproducibility and cross-study comparison. By combining quantitative force measurements with molecular specificity, spatial mapping, and multimodal analysis, AFM-FS is positioned to become an increasingly powerful platform for decoding adhesion phenomena in living, pathogen-associated, and viral interfaces.

## Data Availability

No datasets were generated or analysed during the current study.

## References

[CR1] Abu-Lail NI, Camesano TA (2003) Role of lipopolysaccharides in the adhesion, retention, and transport of *Escherichia coli* JM109. Environ Sci Technol 37:2173–2183. 10.1021/es026159o 12785523

[CR2] Aguayo S, Donos N, Spratt D, Bozec L (2015) Nanoadhesion of Staphylococcus aureus onto titanium implant surfaces. J Dent Res 94:1078–1084. 10.1177/0022034515591485 26130256

[CR3] Aikawa M, Kamanura K, Shiraishi S, Matsumoto Y, Arwati H, Torii M, Ito Y, Takeuchi T, Tandler B (1996) Membrane knobs of unfixed *Plasmodium falciparum* infected erythrocytes: new findings as revealed by atomic force microscopy and surface potential spectroscopy. Exp Parasitol 84:339–343. 10.1006/expr.1996.0122 8948323

[CR4] Alam F, Balani K (2017) Adhesion force of staphylococcus aureus on various biomaterial surfaces. J Mech Behav Biomed Mater 65:872–880. 10.1016/j.jmbbm.2016.10.009 27814559

[CR5] Alsteens D, Pfreundschuh M, Zhang C, Spoerri PM, Coughlin SR, Kobilka BK, Müller DJ (2015) Imaging G protein–coupled receptors while quantifying their ligand-binding free-energy landscape. Nat Methods 12:845–851. 10.1038/nmeth.3479 PMC5087271 26167642

[CR6] Álvarez-López A, Tabraue-Rubio R, Hernández-Escobar S, Daza R, Colchero L, Rezvanian P, Elices M, Guinea GV, González-Nieto D, Pérez-Rigueiro J (2023) Application of single cell force spectroscopy (SCFS) to the assessment of cell adhesion to peptide-decorated surfaces. Int J Biol Macromol 244:125369. 10.1016/j.ijbiomac.2023.125369 37321435

[CR7] Amorim MDSDN, Batista JA, Junior FM, Fontes A, Santos-Oliveira R, Rebelo Alencar LM (2022) New insights into hemolytic anemias: ultrastructural and nanomechanical investigation of red blood cells showed early morphological changes. J Biomed Nanotechnol 18:405–421. 10.1166/jbn.2022.3267 35484760

[CR8] Ando T, Fukuda S, Ngo KX, Flechsig H (2024) High-speed atomic force microscopy for filming protein molecules in dynamic action. Annu Rev Biophys 53:19–39. 10.1146/annurev-biophys-030722-113353 38060998

[CR9] Awassa J, Soulé S, Cornu D, Ruby C, El-Kirat-Chatel S (2024) Understanding the nanoscale adhesion forces between the fungal pathogen Candida albicansand antimicrobial zinc-based layered double hydroxides using single-cell and single-particle force spectroscopy. Nanoscale 16:5383–5394. 10.1039/D3NR06027F 38375749

[CR10] Bally M, Rydell GE, Zahn R, Nasir W, Eggeling C, Breimer ME, Svensson L, Höök F, Larson G (2012) Norovirus GII.4 virus-like particles recognize galactosylceramides in domains of planar supported lipid bilayers. Angew Chem Int Ed 51:12020–12024. 10.1002/anie.201205972 PMC3546384 23097253

[CR11] Banerjee N, Mukhopadhyay S (2016) Viral glycoproteins: biological role and application in diagnosis. VirusDis 27:1–11. 10.1007/s13337-015-0293-5 PMC4758313 26925438

[CR12] Baumgartner W, Hinterdorfer P, Ness W, Raab A, Vestweber D, Schindler H, Drenckhahn D (2000) Cadherin interaction probed by atomic force microscopy. Proc Natl Acad Sci U S A 97:4005–4010. 10.1073/pnas.070052697 PMC18132 10759550

[CR13] Beaussart A, El-Kirat-Chatel S (2019) Microbial adhesion and ultrastructure from the single-molecule to the single-cell levels by atomic force microscopy. Cell Surf 5:100031. 10.1016/j.tcsw.2019.100031 PMC7389263 32743147

[CR14] Beaussart A, Alsteens D, El-Kirat-Chatel S, Lipke PN, Kucharíková S, Van Dijck P, Dufrêne YF (2012) Single-molecule imaging and functional analysis of als adhesins and mannans during Candida albicans morphogenesis. ACS Nano 6:10950–10964. 10.1021/nn304505s PMC3528847 23145462

[CR15] Björnham O, Andersson M (2017) Theory for nonlinear dynamic force spectroscopy. Eur Biophys J 46:225–233. 10.1007/s00249-016-1158-6 PMC5346443 27461369

[CR16] Butt H-J, Cappella B, Kappl M (2005) Force measurements with the atomic force microscope: technique, interpretation and applications. Surf Sci Rep 59:1–152. 10.1016/j.surfrep.2005.08.003

[CR17] Cappella B, Dietler G (1999) Force-distance curves by atomic force microscopy. Surf Sci Rep 34:1–104. 10.1016/S0167-5729(99)00003-5

[CR18] Cardoso-Lima R, Souza PFN, Guedes MIF, Santos-Oliveira R, Rebelo Alencar LM (2021) SARS-CoV-2 unrevealed: ultrastructural and nanomechanical analysis. Langmuir 37:10762–10769. 10.1021/acs.langmuir.1c01488 34351770

[CR19] Cardoso-Lima R, Filho JFSD, De Araujo Dorneles ML, Gaspar RS, Souza PFN, Costa Dos Santos C, Santoro Rosa D, Santos-Oliveira R, Alencar LMR (2022) Nanomechanical and vibrational signature of chikungunya viral particles. Viruses 14:2821. 10.3390/v14122821 PMC9782469 36560825

[CR20] Cardoso-Lima R, Santos-Oliveira R, Souza PFN, Barbosa LRS, Wuite GJL, Alencar LMR (2023) Physical virology: how physics is enabling a better understanding of recent viral invaders. Biophys Rev 15:611–623. 10.1007/s12551-023-01075-4 PMC10480132 37681101

[CR21] Carvalho PA, Diez-Silva M, Chen H, Dao M, Suresh S (2013) Cytoadherence of erythrocytes invaded by Plasmodium falciparum: Quantitative contact-probing of a human malaria receptor. Acta Biomater 9:6349–6359. 10.1016/j.actbio.2013.01.019 PMC3870279 23376131

[CR22] Casasnovas JM (2013) Virus-receptor interactions and receptor-mediated virus entry into host cells. In: Mateu MG (ed) Structure and physics of viruses, subcellular biochemistry. Springer Netherlands, Dordrecht, pp 441–466. 10.1007/978-94-007-6552-8_15 PMC7122110 23737061

[CR23] Chala N, Zhang X, Zambelli T, Zhang Z, Schneider T, Panozzo D, Poulikakos D, Ferrari A (2023) 4D force detection of cell adhesion and contractility. Nano Lett 23:2467–2475. 10.1021/acs.nanolett.2c03733 PMC10103301 36975035

[CR24] Chirasatitsin S, Engler AJ (2010) Detecting cell-adhesive sites in extracellular matrix using force spectroscopy mapping. J Phys Condens Matter 22:194102. 10.1088/0953-8984/22/19/194102 PMC2997741 21152375

[CR25] Cho DH, Aguayo S, Cartagena-Rivera AX (2023) Atomic force microscopy-mediated mechanobiological profiling of complex human tissues. Biomaterials 303:122389. 10.1016/j.biomaterials.2023.122389 PMC10842832 37988897

[CR26] Da Costa TM, Viljoen A, Towell AM, Dufrêne YF, Geoghegan JA (2022) Fibronectin binding protein B binds to loricrin and promotes corneocyte adhesion by Staphylococcus aureus. Nat Commun 13:2517. 10.1038/s41467-022-30271-1 PMC9076634 35523796

[CR27] De Araujo Dorneles ML, Cardoso-Lima R, Souza PFN, Santoro Rosa D, Magne TM, Santos-Oliveira R, Alencar LMR (2022) Zika Virus (ZIKV): a new perspective on the nanomechanical and structural properties. Viruses 14:1727. 10.3390/v14081727 PMC9414353 36016349

[CR28] De Pablo PJ, Schaap IAT (2019) Atomic force microscopy of viruses. In: Greber UF (ed) Physical virology, advances in experimental medicine and biology. Springer International Publishing, Cham, pp 159–179. 10.1007/978-3-030-14741-9_8 31317500

[CR29] Delguste M, Brun GL, Cotin F, Machiels B, Gillet L, Alsteens D (2021) Single-virus force spectroscopy discriminates the intrinsic role of two viral glycoproteins upon cell surface attachment. Nano Lett 21:847–853. 10.1021/acs.nanolett.0c04609 33373240

[CR30] Deng X, Xiong F, Li X, Xiang B, Li Z, Wu X, Guo C, Li X, Li Y, Li G, Xiong W, Zeng Z (2018) Application of atomic force microscopy in cancer research. J Nanobiotechnol 16:102. 10.1186/s12951-018-0428-0 PMC6288943 30538002

[CR31] Derjaguin BV, Muller VM, Toporov YP (1975) Effect of contact deformations on the adhesion of particles. J Colloid Interface Sci 53:314–326. 10.1016/0021-9797(75)90018-1

[CR32] Dias Rates ER, Almeida CD, De Paula Fiod Costa E, Jansen De Mello Farias R, Santos-Oliveira R, Rebelo Alencar LM (2024) Evaluation of biophysical alterations in the epithelial and endothelial layer of patients with Bullous Keratopathy. Exp Eye Res 240:109791. 10.1016/j.exer.2024.109791 38253307

[CR33] Do Nascimento Amorim MDS, Rates ERD, Isabela Vitoria de ACM, Silva Diniz Filho JF, dos Santos CC, Santos-Oliveira R, Simões Gaspar R, Rodrigues Sanches J, Araújo Serra Pinto B, de Andrade Paes AM, Alencar LMR (2024) Diabetes and cognitive decline: an innovative approach to analyzing the biophysical and vibrational properties of the hippocampus. ACS Omega. 10.1021/acsomega.4c05869 PMC11447714 39371966

[CR34] Dos Santos Natividade R, Danzer B, Somoza V, Koehler M (2025) Atomic force microscopy at the forefront: unveiling foodborne viruses with biophysical tools. npj Viruses 3, 25. 10.1038/s44298-025-00107-y PMC11971264 40295860

[CR35] Ducker WA, Senden TJ, Pashley RM (1991) Direct measurement of colloidal forces using an atomic force microscope. Nature 353:239–241. 10.1038/353239a0

[CR36] Dufrêne YF (2008) Towards nanomicrobiology using atomic force microscopy. Nat Rev Microbiol 6:674–680. 10.1038/nrmicro1948 18622407

[CR37] Dufrêne YF (2015) Sticky microbes: forces in microbial cell adhesion. Trends Microbiol 23:376–382. 10.1016/j.tim.2015.01.011 25684261

[CR38] Dufrêne YF, Martínez-Martín D, Medalsy I, Alsteens D, Müller DJ (2013) Multiparametric imaging of biological systems by force-distance curve–based AFM. Nat Methods 10:847–854. 10.1038/nmeth.2602 23985731

[CR39] Dufrêne YF, Ando T, Garcia R, Alsteens D, Martinez-Martin D, Engel A, Gerber C, Müller DJ (2017) Imaging modes of atomic force microscopy for application in molecular and cell biology. Nat Nanotechnol 12:295–307. 10.1038/nnano.2017.45 28383040

[CR40] Dufrêne YF (2014) Atomic force microscopy in microbiology: new structural and functional insights into the microbial cell surface. mBio 5:e01363–14. 10.1128/mBio.01363-14 PMC4120197 25053785

[CR41] Evans E (2001) Probing the relation between force—lifetime—and chemistry in single molecular bonds. Annu Rev Biophys Biomol Struct 30:105–128. 10.1146/annurev.biophys.30.1.105 11340054

[CR42] Evans E, Ritchie K (1997) Dynamic strength of molecular adhesion bonds. Biophys J 72:1541–1555. 10.1016/S0006-3495(97)78802-7 PMC1184350 9083660

[CR43] Fang HHP, Chan K-Y, Xu L-C (2000) Quantification of bacterial adhesion forces using atomic force microscopy (AFM). J Microbiol Methods 40:89–97. 10.1016/S0167-7012(99)00137-2 10739347

[CR44] Feuillie C, Vitry P, McAleer MA, Kezic S, Irvine AD, Geoghegan JA, Dufrêne YF (2018) Adhesion of *Staphylococcus aureus* to corneocytes from atopic dermatitis patients is controlled by natural moisturizing factor levels. mBio 9:e01184–18. 10.1128/mBio.01184-18 PMC6094479 30108169

[CR45] Formosa-Dague C, Fu Z-H, Feuillie C, Derclaye S, Foster TJ, Geoghegan JA, Dufrêne YF (2016) Forces between Staphylococcus aureus and human skin. Nanoscale Horiz 1:298–303. 10.1039/C6NH00057F 32260649

[CR46] Friedrichs J, Legate KR, Schubert R, Bharadwaj M, Werner C, Müller DJ, Benoit M (2013) A practical guide to quantify cell adhesion using single-cell force spectroscopy. Methods 60:169–178. 10.1016/j.ymeth.2013.01.006 23396062

[CR47] Gao L, Zhao H, Li T, Huo P, Chen D, Liu B (2018) Atomic force microscopy based tip-enhanced Raman spectroscopy in biology. Int J Mol Sci 19:1193. 10.3390/ijms19041193 PMC5979470 29652860

[CR48] Garcia R, Herruzo ET (2012) The emergence of multifrequency force microscopy. Nat Nanotechnol 7:217–226. 10.1038/nnano.2012.38 22466857

[CR49] Guillaume-Gentil O, Potthoff E, Ossola D, Franz CM, Zambelli T, Vorholt JA (2014) Force-controlled manipulation of single cells: from AFM to FluidFM. Trends Biotechnol 32:381–388. 10.1016/j.tibtech.2014.04.008 24856959

[CR50] Harjumäki R, Zhang X, Nugroho RWN, Farooq M, Lou Y-R, Yliperttula M, Valle-Delgado JJ, Österberg M (2020) AFM force spectroscopy reveals the role of integrins and their activation in cell-biomaterial interactions. ACS Appl Bio Mater 3:1406–1417. 10.1021/acsabm.9b01073 35021633

[CR51] Helenius J, Heisenberg C-P, Gaub HE, Muller DJ (2008) Single-cell force spectroscopy. J Cell Sci 121:1785–1791. 10.1242/jcs.030999 18492792

[CR52] Helms G, Dasanna AK, Schwarz US, Lanzer M (2016) Modeling cytoadhesion of *Plasmodium falciparum-* infected erythrocytes and leukocytes—common principles and distinctive features. FEBS Lett 590:1955–1971. 10.1002/1873-3468.12142 PMC5071704 26992823

[CR53] Hinterdorfer P, Dufrêne YF (2006) Detection and localization of single molecular recognition events using atomic force microscopy. Nat Methods 3:347–355. 10.1038/nmeth871 16628204

[CR54] Hinterdorfer P, Baumgartner W, Gruber HJ, Schilcher K, Schindler H (1996) Detection and localization of individual antibody-antigen recognition events by atomic force microscopy. Proc Natl Acad Sci USA 93:3477–3481. 10.1073/pnas.93.8.3477 PMC39634 8622961

[CR55] Hoffmann T, Dougan L (2012) Single molecule force spectroscopy using polyproteins. Chem Soc Rev 41:4781. 10.1039/c2cs35033e 22648310

[CR56] Holuigue H, Nacci L, Di Chiaro P, Chighizola M, Locatelli I, Schulte C, Alfano M, Diaferia GR, Podestà A (2023) Native extracellular matrix probes to target patient- and tissue-specific cell–microenvironment interactions by force spectroscopy. Nanoscale 15:15382–15395. 10.1039/D3NR01568H 37700706

[CR57] Hughes ML, Dougan L (2016) The physics of pulling polyproteins: a review of single molecule force spectroscopy using the AFM to study protein unfolding. Rep Prog Phys 79:076601. 10.1088/0034-4885/79/7/076601 27309041

[CR58] Ingber DE (2008) Can cancer be reversed by engineering the tumor microenvironment? Semin Cancer Biol 18:356–364. 10.1016/j.semcancer.2008.03.016 PMC2570051 18472275

[CR59] Israelachvili JN (2011) Intermolecular and surface forces, 3rd ed. Academic Press, Burlington, MA

[CR60] Jackson S, Coulthwaite L, Loewy Z, Scallan A, Verran J (2014) Biofilm development by blastospores and hyphae of *Candida albicans* on abraded denture acrylic resin surfaces. J Prosthet Dent 112:988–993. 10.1016/j.prosdent.2014.02.003 24726593

[CR61] James SA, Hilal N, Wright CJ (2017) Atomic force microscopy studies of bioprocess engineering surfaces – imaging, interactions and mechanical properties mediating bacterial adhesion. Biotechnol J 12:1600698. 10.1002/biot.201600698 28488793

[CR62] Johnson KL, Kendall K, Roberts AD (1971) Surface energy and the contact of elastic solids. Proc R Soc Lond A Math Phys Sci 324:301–313. 10.1098/rspa.1971.0141

[CR63] Kannan A, Gautam P (2015) A quantitative study on the formation of *Pseudomonas aeruginosa* biofilm. Springerplus 4:379. 10.1186/s40064-015-1029-0 PMC4516152 26240777

[CR64] Kerdegari S, Canepa P, Odino D, Oropesa-Nuñez R, Relini A, Cavalleri O, Canale C (2023) Insights in cell biomechanics through atomic force microscopy. Materials 16:2980. 10.3390/ma16082980 PMC10142950 37109816

[CR65] Khan N, Aslan H, Büttner H, Rohde H, Golbek TW, Roeters SJ, Woutersen S, Weidner T, Meyer RL (2022) The giant staphylococcal protein Embp facilitates colonization of surfaces through Velcro-like attachment to fibrillated fibronectin. Elife 11:e76164. 10.7554/eLife.76164 PMC9302970 35796649

[CR66] Morris VJ, Kirby AR, Gunning AP (1999) Atomic force microscopy for biologists. Imperial College Press, London

[CR67] Korayem MH, Mehrabani M (2024) Experimental investigation of adhesion force, folding factor and surface roughness of chronic lymphocytic leukemia cell using AFM. Powder Technol 444:120025. 10.1016/j.powtec.2024.120025

[CR68] Krieg M, Fläschner G, Alsteens D, Gaub BM, Roos WH, Wuite GJL, Gaub HE, Gerber C, Dufrêne YF, Müller DJ (2018) Atomic force microscopy-based mechanobiology. Nat Rev Phys 1:41–57. 10.1038/s42254-018-0001-7

[CR69] Kuznetsov YG, McPherson A (2011) Atomic force microscopy in imaging of viruses and virus-infected cells. Microbiol Mol Biol Rev 75:268–285. 10.1128/MMBR.00041-10 PMC3122623 21646429

[CR70] Lehenkari PP, Horton MA (1999) Single integrin molecule adhesion forces in intact cells measured by atomic force microscopy. Biochem Biophys Res Commun 259:645–650. 10.1006/bbrc.1999.0827 10364472

[CR71] Li F, Redick SD, Erickson HP, Moy VT (2003) Force measurements of the α5β1 integrin-fibronectin interaction. Biophys J 84:1252–1262. 10.1016/S0006-3495(03)74940-6 PMC1302701 12547805

[CR72] Li A, Lim TS, Shi H, Yin J, Tan SJ, Li Z, Low BC, Tan KSW, Lim CT (2011) Molecular mechanistic insights into the endothelial receptor mediated cytoadherence of plasmodium falciparum-infected erythrocytes. PLoS ONE 6:e16929. 10.1371/journal.pone.0016929 PMC3060092 21437286

[CR73] Lima RC, Soares REP, Maia Jr FF, Menezes AS, Costa EF, Alencar LMR (2019) Marcadores de superfície para células deleucemias mieloblásticas: uma proposta através da microscopia de força atômica. In: Anais do Encontro Anual daBiofísica 2019. Blucher, São Paulo, pp 87–90. 10.5151/biofisica2019-25

[CR74] Lopes CS, Curty J, Carvalho FA, Hernández-Machado A, Kinoshita K, Santos NC, Travasso RDM (2023) A mathematical model of fibrinogen-mediated erythrocyte–erythrocyte adhesion. Commun Biol 6:192. 10.1038/s42003-023-04560-4 PMC9938206 36801914

[CR75] Marko JF, Siggia ED (1995) Statistical mechanics of supercoiled DNA. Phys Rev E 52:2912–2938. 10.1103/PhysRevE.52.2912 9963738

[CR76] Masud N, Rade J, Hasib MHH, Krishnamurthy A, Sarkar A (2024) Machine learning approaches for improving atomic force microscopy instrumentation and data analytics. Front Phys 12:1347648

[CR77] Mathelié-Guinlet M, Viela F, Viljoen A, Dehullu J, Dufrêne YF (2019) Single-molecule atomic force microscopy studies of microbial pathogens. Curr Opin Biomed Eng 12:1–7. 10.1016/j.cobme.2019.08.001

[CR78] Mierke CT (2024) Extracellular matrix cues regulate mechanosensing and mechanotransduction of cancer cells. Cells 13:96. 10.3390/cells13010096 PMC10777970 38201302

[CR79] Müller DJ, Dufrêne YF (2008) Atomic force microscopy as a multifunctional molecular toolbox in nanobiotechnology. Nat Nanotechnol 3:261–269. 10.1038/nnano.2008.100 18654521

[CR80] Müller DJ, Helenius J, Alsteens D, Dufrêne YF (2009) Force probing surfaces of living cells to molecular resolution. Nat Chem Biol 5:383–390. 10.1038/nchembio.181 19448607

[CR81] Müller DJ, Dumitru AC, Lo Giudice C, Gaub HE, Hinterdorfer P, Hummer G, De Yoreo JJ, Dufrêne YF, Alsteens D (2021) Atomic force microscopy-based force spectroscopy and multiparametric imaging of biomolecular and cellular systems. Chem Rev 121:11701–11725. 10.1021/acs.chemrev.0c00617 33166471

[CR82] Nagao E, Kaneko O, Dvorak JA (2000) *Plasmodium falciparum*-infected erythrocytes: qualitative and quantitative analyses of parasite-induced knobs by atomic force microscopy. J Struct Biol 130:34–44. 10.1006/jsbi.2000.4236 10806089

[CR83] Ogharandukun E, Tewolde W, Damtae E, Wang S, Ivanov A, Kumari N, Nekhai S, Chandran PL (2020) Establishing rules for self-adhesion and aggregation of N-glycan sugars using virus glycan shields. Langmuir 36:13769–13783. 10.1021/acs.langmuir.0c01953 PMC7798417 33186493

[CR84] Okajima T, Kuribayashi-Shigetomi K (2023) Atomic force microscopy for investigating cell and tissue mechanics as heterogeneous and hierarchical materials. J Biomech Sci Eng 18:23–00339

[CR85] Ovchinnikova ES, Krom BP, Busscher HJ, Van Der Mei HC (2012) Evaluation of adhesion forces of Staphylococcus aureus along the length of Candida albicanshyphae. BMC Microbiol 12:281. 10.1186/1471-2180-12-281 PMC3538519 23181353

[CR86] Perez-Guaita D, Kochan K, Batty M, Doerig C, Garcia-Bustos J, Espinoza S, McNaughton D, Heraud P, Wood BR (2018) Multispectral atomic force microscopy-infrared nano-imaging of malaria infected red blood cells. Anal Chem 90:3140–3148. 10.1021/acs.analchem.7b04318 29327915

[CR87] Prats-Mateu B, Gierlinger N (2017) Tip in–light on: advantages, challenges, and applications of combining AFM and Raman microscopy on biological samples. Microsc Res Tech 80:30–40. 10.1002/jemt.22744 PMC5217061 27514318

[CR88] Puech P-H, Poole K, Knebel D, Muller DJ (2006) A new technical approach to quantify cell–cell adhesion forces by AFM. Ultramicroscopy 106:637–644. 10.1016/j.ultramic.2005.08.003 16675123

[CR89] Quaroni L, Pogoda K, Zuber J-W, Kwiatek W (2017) Mid-infrared spectroscopy and microscopy of subcellular structures in eukaryotic cells with atomic force microscopy - infrared spectroscopy. 10.48550/ARXIV.1704.01395 PMC9077331 35541450

[CR90] Rahimi E, Palacios‐Corella M, Mol A, Pané S, Puigmartí‐Luis J (2025) Kelvin probe force microscopy in bionanotechnology: current advances and future perspectives. Adv Mater. 10.1002/adma.202510671 PMC13569047 40904270

[CR91] Ray A, Simpson JD, Demir I, Gisbert VG, Gomes DB, Amadei F, Alsteens D (2025) From viral assembly to host interaction: AFM’s contributions to virology. J Virol 99:e00873-24. 10.1128/jvi.00873-24 PMC11784315 39655953

[CR92] Rojas FP, Batista MA, Lindburg CA, Dean D, Grodzinsky AJ, Ortiz C, Han L (2014) Molecular adhesion between cartilage extracellular matrix macromolecules. Biomacromol 15:772–780. 10.1021/bm401611b PMC3983133 24491174

[CR93] Rousso I, Deshpande A (2022) Applications of atomic force microscopy in HIV-1 research. Viruses 14:648. 10.3390/v14030648 PMC8955997 35337055

[CR94] Scholz K, Papagrigorakes M, Lettermann L, Pennarola F, Patra P, Dasanna AK, Sanchez CP, Kehrer J, Cavalcanti-Adam EA, Schwarz US, Tanaka M, Lanzer M (2025) Contact dynamics of cytoadhering *Plasmodium falciparum*-infected erythrocytes in flow. ACS Infect Dis 11:2628–2643. 10.1021/acsinfecdis.5c00594 PMC12442085 40783841

[CR95] Silva França AR, Diniz-Filho JFS, Dos Santos CC, Durço Coimbra L, Marques RE, Barbosa LRS, Santos-Oliveira R, Souza PFN, Alencar LMR (2024) Unraveling the nanomechanical and vibrational properties of the mayaro virus. ACS Omega 9:48397–48404. 10.1021/acsomega.4c06749 PMC11635520 39676942

[CR96] Simpson JD, Ray A, Marcon C, Dos Santos Natividade R, Dorrazehi GM, Durlet K, Koehler M, Alsteens D (2023) Single-molecule analysis of SARS-CoV-2 binding to C-type lectin receptors. Nano Lett 23:1496–1504. 10.1021/acs.nanolett.2c04931 PMC9924085 36758952

[CR97] Sotres J, Arnebrant T (2013) Experimental investigations of biological lubrication at the nanoscale: the cases of synovial joints and the oral cavity. Lubricants 1:102–131. 10.3390/lubricants1040102

[CR98] Suenaga T, Arase H (2015) Viral interactions with glycans. In: Taniguchi N, Endo T, Hart GW, Seeberger PH, Wong C-H (eds) Glycoscience: biology and medicine. Springer Japan, Tokyo, pp 785–794. 10.1007/978-4-431-54841-6_152

[CR99] Sullan RMA, Beaussart A, Tripathi P, Derclaye S, El-Kirat-Chatel S, Li JK, Schneider Y-J, Vanderleyden J, Lebeer S, Dufrêne YF (2014) Single-cell force spectroscopy of pili-mediated adhesion. Nanoscale 6:1134–1143. 10.1039/C3NR05462D 24296882

[CR100] Sun M, Graham JS, Hegedüs B, Marga F, Zhang Y, Forgacs G, Grandbois M (2005) Multiple membrane tethers probed by atomic force microscopy. Biophys J 89:4320–4329. 10.1529/biophysj.104.058180 PMC1366996 16183875

[CR101] Thomas-Chemin O, Janel S, Boumehdi Z, Séverac C, Trevisiol E, Dague E, Duprés V (2025) Advancing high-throughput cellular atomic force microscopy with automation and artificial intelligence. ACS Nano 19:5045–5062 10.1021/acsnano.4c07729 39883411

[CR102] V. D. Dos Santos AC, Hondl N, Ramos-Garcia V, Kuligowski J, Lendl B, Ramer G (2023) AFM-IR for nanoscale chemical characterization in life sciences: recent developments and future directions. ACS Meas Sci Au 3:301–314. 10.1021/acsmeasuresciau.3c00010 PMC10588935 37868358

[CR103] Valle-Delgado JJ, Urbán P, Fernàndez-Busquets X (2013) Demonstration of specific binding of heparin to *Plasmodium falciparum*-infected vs. non-infected red blood cells by single-molecule force spectroscopy. Nanoscale 5:3673. 10.1039/c2nr32821f 23306548

[CR104] Van Roy F (2014) Beyond E-cadherin: roles of other cadherin superfamily members in cancer. Nat Rev Cancer 14:121–134. 10.1038/nrc3647 24442140

[CR105] Viljoen A, Mathelié-Guinlet M, Ray A, Strohmeyer N, Oh YJ, Hinterdorfer P, Müller DJ, Alsteens D, Dufrêne YF (2021) Force spectroscopy of single cells using atomic force microscopy. Nat Rev Methods Primers 1:63. 10.1038/s43586-021-00062-x

[CR106] Waite JR, Tan SY, Saha H, Sarkar S, Sarkar A (2023) Few-shot deep learning for AFM force curve characterization of single-molecule interactions. Patterns (n y). 10.1016/j.patter.2022.100672 PMC9868661 36699737

[CR107] Wang Y, Yuan H, Miao Z, Liu J, Yang X, Wang Y (2025) Molecular Velcro for characterizing hydrogel coating adhesion. Extreme Mech Lett 77:102357. 10.1016/j.eml.2025.102357

[CR108] Wei J, Yang Y, Li M (2024) Single-cell force spectroscopy of fluid flow-tuned cell adhesion for dissecting hemodynamics in tumor metastasis. Nanoscale 16:360–372. 10.1039/D3NR04439D 38063483

[CR109] Xiao J, Dufrêne YF (2016) Optical and force nanoscopy in microbiology. Nat Microbiol 1:16186. 10.1038/nmicrobiol.2016.186 PMC5839876 27782138

[CR110] Yakovenko O, Sharma S, Forero M, Tchesnokova V, Aprikian P, Kidd B, Mach A, Vogel V, Sokurenko E, Thomas WE (2008) FimH forms catch bonds that are enhanced by mechanical force due to allosteric regulation. J Biol Chem 283:11596–11605. 10.1074/jbc.M707815200 PMC2431072 18292092

[CR111] Yang J, Petitjean SJL, Koehler M, Zhang Q, Dumitru AC, Chen W, Derclaye S, Vincent SP, Soumillion P, Alsteens D (2020) Molecular interaction and inhibition of SARS-CoV-2 binding to the ACE2 receptor. Nat Commun 11:4541. 10.1038/s41467-020-18319-6 PMC7486399 32917884

[CR112] Zeng W, Jia X, Chi X, Zhang X, Li E, Wu Y, Liu Y, Han J, Ni K, Ye X, Hu X, Ma H, Yu C, Chiu S, Jin T (2023) An engineered bispecific nanobody in tetrameric secretory IgA format confers broad neutralization against SARS-CoV-1&2 and most variants. Int J Biol Macromol 253:126817. 10.1016/j.ijbiomac.2023.126817 37690653

[CR113] Zhang K, Reeves CL, Moghaddar M, Fox KE, Elbourne A, Gelmi A (2025) At the pointy end of mechanobiology: AFM for transient biomechanical analysis. Adv Healthc Mater 14:e02026 10.1002/adhm.202502026 PMC12683222 40847732

[CR114] Zhou L, Cai M, Tong T, Wang H (2017) Progress in the correlative atomic force microscopy and optical microscopy. Sensors (Basel) 17:938. 10.3390/s17040938 PMC5426934 28441775

